# 2D Indium Oxide at the Epitaxial Graphene/SIC Interface: Synthesis, Structure, Properties, and Devices

**DOI:** 10.1002/adma.202516133

**Published:** 2025-11-10

**Authors:** Furkan Turker, Bohan Xu, Chengye Dong, Michael Labella, Nadire Nayir, Natalya Sheremetyeva, Zachary J. Trdinich, Duanchen Zhang, Gokay Adabasi, Bita Pourbahari, Li‐Syuan Lu, Wesley E. Auker, Ke Wang, Mehmet Baykara, Vincent Meunier, Nabil Bassim, Adri C. T. van Duin, Vincent H. Crespi, Joshua A. Robinson

**Affiliations:** ^1^ Department of Materials Science and Engineering The Pennsylvania State University University Park PA 16802 USA; ^2^ Center for 2‐Dimensional and Layered Materials The Pennsylvania State University University Park PA 16802 USA; ^3^ Department of Physics The Pennsylvania State University University Park PA 16802 USA; ^4^ Two‐Dimensional Crystal Consortium The Pennsylvania State University University Park PA 16802 USA; ^5^ Materials Research Institute The Pennsylvania State University University Park PA 16802 USA; ^6^ Paul‐Drude‐Institute for Solid State Electronics Leibniz Institute within Forschungsverbund Berlin eV. Hausvogteiplatz, 5‐7 10117 Berlin Germany; ^7^ Department of Physics Engineering Istanbul Technical University Maslak Istanbul 34469 Turkey; ^8^ Department of Mechanical Engineering The Pennsylvania State University University Park PA 16802 USA; ^9^ Department of Engineering Science and Mechanics The Pennsylvania State University University Park PA 16802 USA; ^10^ Department of Mechanical Engineering University of California Merced Merced CA 95343 USA; ^11^ Department of Materials Science and Engineering McMaster University Hamilton Ontario L8S 4L8 Canada; ^12^ Canadian Centre for Electron Microscopy Hamilton Ontario L8S 4L8 Canada; ^13^ Department of Chemistry The Pennsylvania State University University Park PA 16802 USA

**Keywords:** 2D indium oxide, graphene, heterostructure, intercalation, vertical Schottky diode

## Abstract

Scaled and high‐quality insulators are crucial for fabricating 2D/3D hybrid vertical electronic devices such as metal‐oxide‐semiconductor (MOS) based Schottky diodes and hot electron transistors, the production of which is constrained by the scarcity of bulk layered wide bandgap semiconductors. In this research, the synthesis of a new 2D insulator, monolayer InO_2_, which differs in stoichiometry from its bulk form is presented, over a large area (>300 µm^2^) by intercalating at the epitaxial graphene (EG)/SiC interface. By adjusting the lateral size of graphene through optical lithography prior to the intercalation, the thickness of InO_2_ is tuned such that it is 85% monolayer. The preference for monolayer formation of InO_2_ is explained using molecular dynamics and density functional theory (DFT) calculations. Additionally, the bandgap of InO_2_ is calculated to be 4.1 eV, differing from its bulk form (2.7 eV). Furthermore, MOS‐based Schottky diode measurements on InO_2_ intercalated EG/n‐SiC demonstrate that the EG/n‐SiC junction transforms from ohmic to a Schottky junction upon intercalation, with a barrier height of 0.87 eV and a rectification ratio of ≈10^5^. These findings introduce a new addition to the 2D insulator family, demonstrating the utility of monolayer InO_2_ as a barrier in vertical electronic devices.

## Graphical abstract

A new monolayer insulator, InO_2_, is synthesized by intercalating indium beneath patterned epitaxial graphene on SiC, followed by high‐temperature oxidation. This selective confinement yields large‐area, uniform InO_2_ with a wide bandgap of 4.1 eV. Upon intercalation, the EG/n‐SiC junction transitions from ohmic to Schottky, exhibiting a rectification ratio of ≈10⁴—promising for high‐frequency vertical electronic devices.

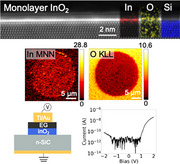

## 1 Introduction

Confining species at the interface between epitaxial graphene (EG) and silicon carbide (SiC) paved the way for the creation of large‐scale, stable monolayer to few‐layer metals,^[^ ^1^ ^]^ metal alloys,^[^ ^2^ ^]^ and metal compounds.^[^ ^3^, ^4^ ^]^ The confinement of these metallic structures at this distinctive asymmetric interface has led to the development of several unique properties, including metal‐to‐semiconductor transitions^[^ ^5^ ^]^ and superconductivity.^[^ ^1^ ^]^ This enables opportunities to explore 2D insulators, a field constrained by the narrow range of available wide bandgap semiconductors that are bulk and layered. An early, exemplary paper is the encapsulation of 2D‐GaN_x_^[^ ^4^ ^]^ (noted for its bandgap of ≈5 eV)— since then, the intercalation of wide and ultra‐wide bandgap compounds, such as AlN_x_,^[^ ^6^ ^]^ GaN_x_,^[^ ^7^ ^]^ InO_x_,^[^ ^8^ ^]^ GaO_x_,^[^ ^3^ ^]^ have been demonstrated.

Insulator intercalation at the EG/SiC interface offers a foundation for developing 2D/3D hybrid vertical electronic devices such as metal‐oxide‐semiconductor (MOS)‐based Schottky diodes and hot electron transistors (HETs). Previous HET studies utilizing EG/SiC, generally report poor output characteristics and a low on‐off ratio due to high leakage current through the as‐grown EG/SiC junction.^[^ ^9^ ^]^ The ohmic behavior of the EG/SiC (0001) interface can be converted into a Schottky junction through hydrogen intercalation.^[^ ^10^ ^]^ This transformation results from the differences in graphene's work function and the electron affinity of SiC as well as the spontaneous polarization of hexagonal SiC. The reported barrier height at the hydrogen‐intercalated EG/SiC (0001) interface varies significantly from 0.8 to 1.6 eV, indicating that the conditions of graphene growth and hydrogen intercalation are crucial parameters for controlling electron transport across the EG/SiC interface.^[^ ^10^, ^11^, ^12^ ^]^ An alternative method for the EG/SiC based Schottky diode formation is via insulator intercalation, which yields a higher threshold voltage (Vth) and rectification ratio (RR) than H‐intercalated.^[^ ^3^, ^4^, ^8^ ^]^

Building on these advancements, the formation of 2D indium oxide (InO_x_) through intercalation is particularly appealing due to its moderate bandgap (≈2.7 eV) and ability for indium (In) to be intercalated over a large area.^[^ ^1^ ^]^ Common techniques used to produce ultrathin InO_x_, such as atomic layer deposition (ALD)^[^ ^13^ ^]^ and printing an oxide skin from liquid metal,^[^ ^14^ ^]^ typically yield large area coverage but result in poor crystallinity due to low temperature annealing (<250 °C). Therefore, intercalation at the EG/SiC interface offer a viable approach for achieving highly crystalline, ultrathin InO_x_ epitaxial to SiC substrate. Based on our knowledge, there is only one study experimentally demonstrating the intercalation of 2D InO_x_ (bilayer) at the EG/SiC interface where In(CH_3_)_3_ and trace amount of impurities (H_2_O and O_2_) in the gas stream were used as In and O precursors via metal–organic chemical vapor deposition (MOCVD).^[^ ^8^ ^]^ Although Schottky barrier formation upon InO_x_ intercalation was verified via conductive atomic force microscopy (C‐AFM), practical device implementation was hindered by the small lateral size of the 2D InO_x_.

While significant advancements in the lateral uniformity of metals intercalated at the EG/SiC interface to the centimeter scale (excluding step edges of SiC) have been achieved,^[^ ^1^ ^]^ advancements in compound intercalation have not kept pace. Challenges in large area synthesis persist for oxides and nitrides^[^ ^4^, ^7^ ^]^ due to quasi‐3D growth at the EG/SiC interface, leading to the formation of graphene cracks. A key distinction exists between metal and compound intercalation: metals such as Ga, In, Sn, Ag, and Au exhibit self‐limiting growth^[^ ^1^, ^5^ ^]^ and stabilize at 1–3 layers thick (4.5 Å/7.5 Å for 1/3 layers of Ga) at the EG/SiC interface. On the other hand, compounds, such as GaN_x_, can exceed 15 layers (>7 nm). This, in turn, causes graphene to crack and restricts the lateral dimensions of 2D GaN_x_ to just a few microns.^[^ ^4^, ^15^ ^]^ These observations underscore the importance of developing new or modified synthesis techniques for the compound formation via intercalation.

In this study, we demonstrate that graphene patterning via optical lithography prior to the InO_x_ formation significantly improves the uniformity of 2D InO_x_ layers. This method, dubbed selective area confinement heteroepitaxy (SA‐CHet), allows for the intercalation of InO_x_ over areas as large as 300 µm^2^ at the EG/SiC interface. Moreover, using patterned or continuous graphene results in the formation of monolayer InO_2_ (different stoichiometry than bulk In_2_O_3_) or multilayer InO_x_, respectively. Using high‐resolution scanning transmission electron microscopy (STEM) and theoretical calculations via multi‐physics simulations that combine density functional theory (DFT) and ReaxFF reactive molecular dynamics simulations, we explain the mechanism leading to thickness variation in these experimental setups. We further elucidate the structural and electronic properties of monolayer InO_2_, such as the phonon and electronic band structure, both theoretically through DFT and experimentally using Raman spectroscopy and EELS. Finally, given the large lateral size of 2D InO_2_, a MOS based Schottky diode is fabricated using EG/InO_2_/n‐SiC which exhibits current densities of ≈10^5^ A cm^−2^ and rectification ratios of ≈10^5^.

## 2 Results and Discussion

**Figure 1:**
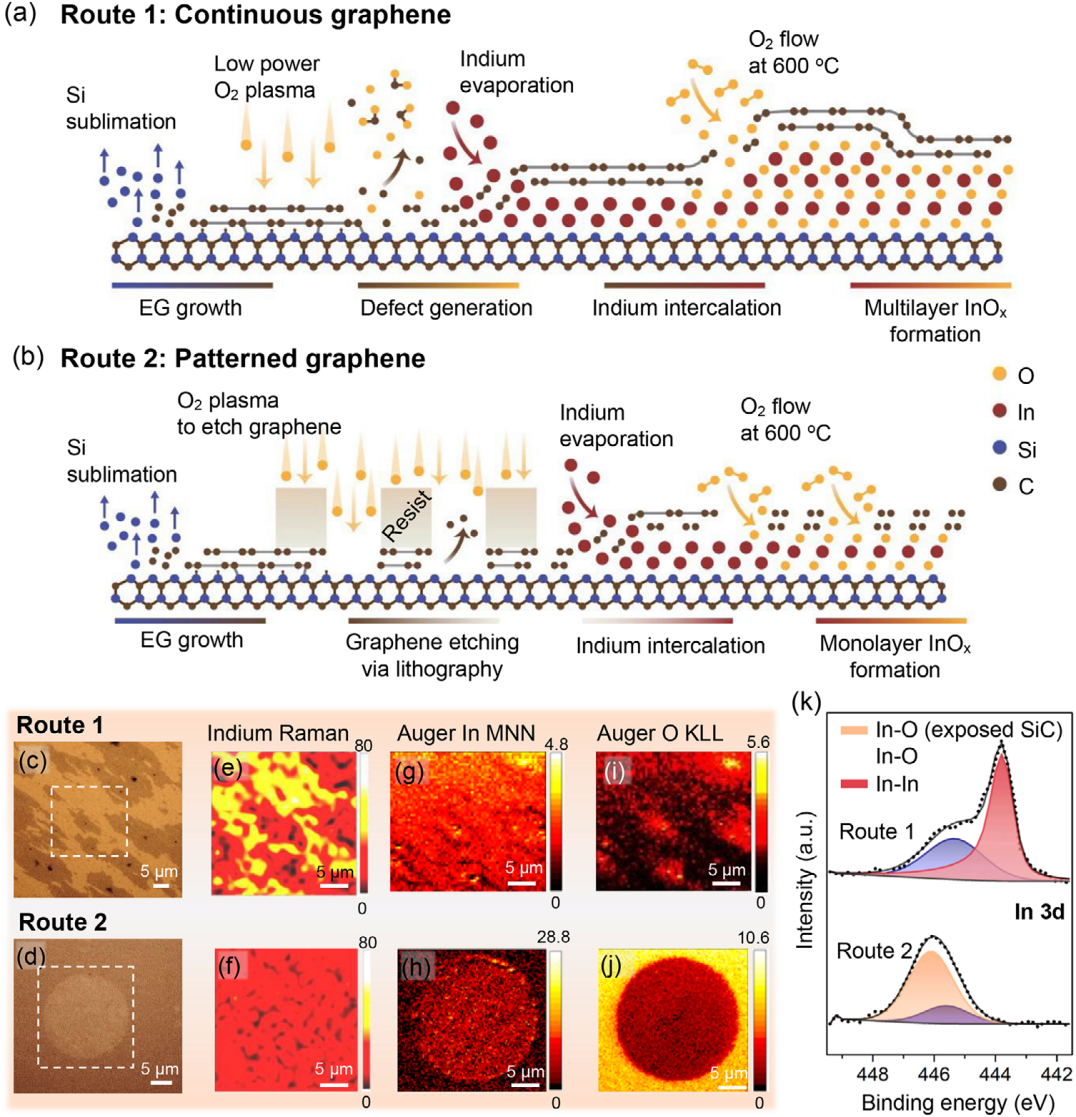
Illustration of the routes for the intercalation of 2D InO_x_ at the EG/SiC interface by using continuous a) and patterned b) graphene. Optical micrographs c,d), Raman maps of metallic In e,f), AES In MNN g,h) and O KLL i,j) maps, and XPS high‐resolution In 3d spectra k) of the In intercalated samples after oxidation at 600 °C, demonstrating that oxidation with continuous graphene (route 1) yields mixed In/InO_x_, while fully oxidized In can be obtained with patterned graphene (route 2).

InO_x_ is formed at the EG/SiC interface using: continuous (route 1) or patterned EG (route 2) (**Figure** **1**). Route 1 employs confinement heteroepitaxy (CHet), wherein continuous EG (1 x 1 cm^2^) undergoes low‐power O2 plasma treatment (50 W) to induce defects, followed by In intercalation, similar to the process described in ref. [1] (Figure 1a; Figure S1, Supporting Information).^[^ ^1^ ^]^ In Route 2, SA‐CHet is employed, where as‐grown EG is first patterned via optical lithography and etched to create predefined graphene circles with 20 µm diameter before In intercalation (Figure 1b). Since In can intercalate through the graphene edges in the pattern, where defects are more prevalent, the O_2_ plasma treatment is unnecessary (Figures S2 and S3, Supporting Information). Post‐In intercalation Raman spectra and mapping, presented in Figure S2 (Supporting Information), show continuous ultra‐low frequency (ULF) metallic In peak (17 cm^−1^), confirming uniform In intercalation.^[^ ^16^ ^]^ Note that thick In particles form beneath the graphene following intercalation, acting as agglomeration sites during this process (Figure S4b,c, Supporting Information). During oxidation, these regions are the first to exhibit graphene cracking, presumably due to high stress, leading to In deintercalation and non‐uniform oxide formation (Figure S4d, Supporting Information). Importantly, the density of these particles decreases with larger graphene pattern size and diminishes entirely when intercalation is conducted using the whole sample (1 × 1 cm^2^). Hence, graphene is initially patterned into large squares (1.5 mm × 1.5 mm) and intercalated with In without plasma treatment (Figure S5, Supporting Information). Subsequently, it is further etched into circles with a 20 µm diameter using an additional lithography step before oxidation. Post‐etching, optical micrographs, and Raman In peak mapping confirm a smoother surface and uniform intercalation (Figure S4f,g, Supporting Information). This method significantly suppresses graphene crack formation during oxidation, as evidenced by scanning electron microscope (SEM) images showing a smoother surface (Figure S4h, Supporting Information). The two‐step etching method is not included in Figure 1b for simplicity.

**Figure 2:**
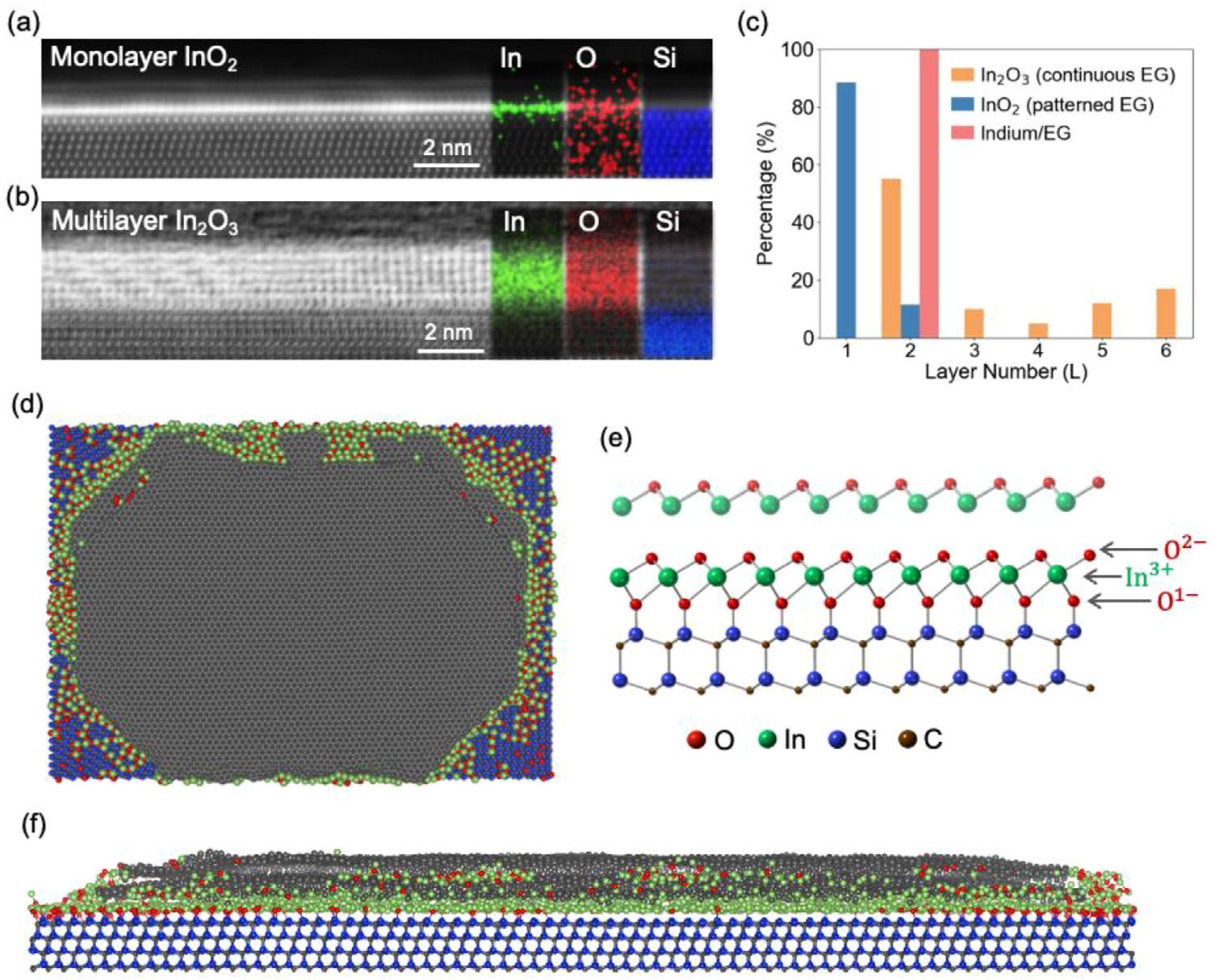
Cross‐sectional HAADF‐STEM images and corresponding EDS maps of monolayer InO_2_ a) and multilayer InO_x_ b) formed using patterned and continuous graphene intercalation, respectively. Thickness analysis of In and InO_x_ intercalated graphene c) measured from STEM images. Top d) and side f) view snapshots acquired from ReaxFF molecular dynamics simulations, showing deintercalation of metal layers (initially two layers) when annealed at 1500 K for 150 ps in an O_2_ flow. Ground state InO_2_ structure e), calculated via DFT, illustrating the stability of monolayer InO_2_ and the detachment of the second InO_x_ layer due to charge imbalance. Si, C, In, and O are represented by blue, brown, green, and red balls, respectively.

The oxidation behavior of 2D In at the EG/SiC interface is dependent on graphene pre‐treatment (e.g., continuous or patterned). In both approaches, samples are oxidized at 600 °C for 30 min under O_2_ flow. Post‐oxidation optical micrograph of the continuous graphene sample (Figure 1c) reveals “bright and dark” contrast regions. Raman mapping (Figure 1e) confirms the bright regions retain metallic In, while Auger electron spectroscopy (AES) mapping (Figure 1i) and X‐Ray photoemission spectroscopy (XPS) (Figure 1k) indicate incomplete oxidation. Additionally, AES In MNN mapping (Figure 1g) indicates In deintercalation from the EG/SiC interface occurs during oxidation. Conversely, post‐oxidation optical micrographs of patterned graphene samples display uniform contrast within the circle (Figure 1d). Raman spectroscopy substantiates the absence of metallic In (Figure 1f), and complete oxidation is confirmed via XPS (Figure 1k). The additional InO_x_ component in the In 3d spectrum (Figure 1k, orange curve) is due to the X‐ray spot size being larger than the circle size is likely to be originated from the InO_x_ particles on the bare SiO_x_/SiC surface or InO_x_ diffused to the SiC substrate during intercalation. Notably, continuous In and oxygen signals are detected in the AES In MNN and O KLL maps (Figure 1h,j), indicating uniform oxidation. The reduced oxygen count inside the circle compared to the bare SiC region (Figure 1j) is due to heavy oxidation of the SiC surface, as these regions lack graphene coverage. Further, AES point scans inside the circle (Figure S6c, Supporting Information) substantiate the successful oxidation of In via graphene patterning. Direct evidence of InO_x_ intercalation is demonstrated in the cross‐sectional STEM image, accompanied by energy dispersive X‐ray spectroscopy (EDS) elemental maps (**Figure** **2**a).

The lateral size of the graphene layer impacts the thickness of intercalated InO_x_ with ≈85% of InO_x_ formed with patterned EG is monolayer (≈6 Å) (Figure 2a,c), while continuous graphene layers yield non‐uniform InO_x_ thicknesses of 2–6 layers thick (Figure 2b,c). STEM analysis indicates metallic In intercalates as a bilayer (Figure S2e, Supporting Information), matching the most stable thickness for In at the EG/SiC interface.^[^ ^1^ ^]^ Reduction in the In thickness to monolayer during oxidation indicates In deintercalation occurs via the edges of patterned graphene, supported by the observation of bulk In_2_O_3_ particles at the perimeter of the circles (Figure S7, Supporting Information). On the other hand, with continuous graphene, In is confined at the interface and can only diffuse laterally beneath EG. This confinement leads to the formation of a broad range of InO_x_ thicknesses (≈11/21 Å for 2/6 L InO_x_), including regions without In (Figures 1, 2). Note that, with continuous graphene samples intercalated with In, we tested In evaporation during oxidation to mitigate indium deintercalation. This approach did not yield good results, likely because the metallic In precursor was oxidized before intercalation. By patterning the graphene via lithography, the diffusion of a second layer of metallic In is facilitated, resulting predominantly in the formation of monolayer InO_x_.

Graphene defect density impacts the In oxidation mechanism. We find that defects generated via O_2_ plasma in continuous graphene are not fully healed during metal intercalation, as the graphene D/G Raman peak ratio is 0.15 ± 0.04, whereas the graphene in patterned samples do not exhibit a measurable Raman D peak (Figures S2b and S6d, Supporting Information). The combination of higher defect density and stress in continuous graphene due to the multilayer InO_x_ formation can cause the graphene to become more prone to oxidation damage at high temperatures, resulting in “holes” in the graphene. As seen from AES In mapping (Figure 1g), this leads to In deintercalation during oxidation, which explains the formation of high‐density bulk In_2_O_3_ particles on the graphene surface and high root‐mean‐square roughness (RMS, R_q_ = 1.99 ± 0.25 nm) (Figure S8, Supporting Information). On the other hand, the RMS roughness of the patterned graphene surface following the oxidation is 4x lower (0.52 ± 0.02 nm). This, along with the presence of bulk In_2_O_3_ particles observed at the perimeter of the circles verifies that the second layer of In can diffuse outward during oxidation when the graphene lateral dimensions are on the order of ≈20 µm.

ReaxFF molecular dynamics simulations confirm that 2D metals tend to diffuse outward in defined graphene structures at elevated temperatures in an O_2_ environment. Here, calculations are conducted using Ga metal, due to the availability of force field data, to provide baseline information for In behavior at the EG/SiC interface. This is possible because In and Ga belong to the same periodic table group and are chemical analogs. As the number of metal layers increases, de‐intercalation becomes more pronounced, resulting in a thinning of the metal layer, particularly near the edges (Figure S9, Supporting Information). Notably, the model with three metal layers shows the highest de‐intercalation rate, leading to a Ga islanding at the center, while only two layers, and eventually one, remain toward the outer edges (Figure S9c,f,i, Supporting Information). Top and side view snapshots from ReaxFF simulations with two layers of Ga are shown in Figure 2d,f, respectively, illustrating Ga deintercalation through the graphene edges. Density functional theory calculations presented in Figure S10 (Supporting Information) reinforce this observation by indicating that the strong covalent interactions between the 1st Ga layer and SiC stabilize the Ga layer at the interface, with a binding energy of 13.63 eV layer^−1^ (Figure S10a, Supporting Information), thereby slowing down Ga diffusion from the edges. However, as additional Ga layers are added, the binding strength between them decreases—the binding energy of the 2nd Ga layer to the 1^st^ is 9.43 eV layer^−1^, and the binding energy of the 3rd Ga layer to the 2nd drops significantly to 6.52 eV layer^−1^. Reduction in binding strength increases the mobility of the metal atoms at the interface, directing them toward the edges where high adsorption sites are located. This outward metal diffusion during oxidation leads to the formation of monolayer InO_2_ with one layer of In and two layers of O as seen in Figure 2a. The In/O atomic ratio calculated from AES is 0.56 ∓ 0.19, supporting an InO_2_ stoichiometry (Figure S6c, Supporting Information).

**Figure 3:**
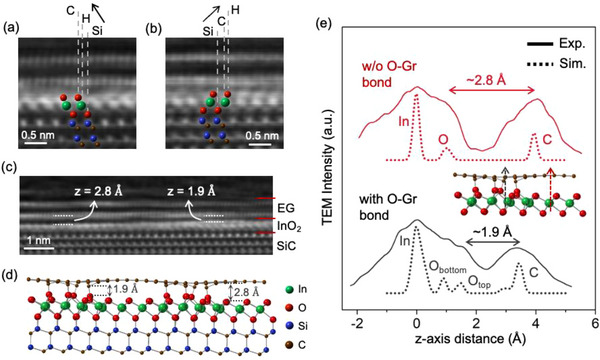
HAADF STEM images of energetically degenerate monolayer InO_2_ structures with Si‐H‐C a) and Si‐C‐H b) stacking sequences. Si, C, and H in (a) and (b) correspond to silicon, carbon, and hollow sites of SiC, respectively. HAADF STEM image showing nanoscale variations in Gr─InO_2_ interlayer distance due to Gr─O bonding c). Ground state, relaxed InO_2_ structure, calculated via DFT, showing intermittent Gr─O bond formation due to In vacancies d). 1.9 and 2.8 Å in (c,d) correspond to interlayer distances between Gr‐InO_2_ with/without Gr─O bonding. Comparison of *z*‐axis STEM intensity profiles of simulated structure in (d) and experimental STEM image in (c) with/without Gr─O bonding, verifying a match for the interlayer distances (e).

Monolayer InO_2_ is thermodynamically preferred at the EG/SiC interface. Cross‐sectional STEM (**Figure** **3**a) provides a foundation for exploring structural relaxations via DFT for monolayer InO_2_ (2L oxygen and 1L In) and bilayer In_2_O_3_ (3L oxygen and 2L In) ‐ initialized at sites projecting onto the silicon (Si), carbon (C), and hollow sites (H) of SiC substrate. The simulated monolayer structure matches experimental observations (Figure 3a), supporting that this is likely the preferred structure because: 1) the detachment of extra layers during structural relaxations of multilayered initial structures and 2) the chemical stability understood by basic chemistry rules and confirmed by free energy comparison against other candidate structures. When initial structures with more than one layer of In and two layers of oxygen are relaxed, two types of outcomes are observed: the extra layer of In and oxygen are detached, leaving two layers of oxygen and one layer of In attached on the SiC substrate (Figure 2e) or some In from the first In layer is ejected such that the original fully‐filled triangular lattice of In becomes 2/3‐filled honeycomb lattice. While multilayer In_2_O_3_ can maintain stability over a certain number of layers when fully confined underneath graphene, it is polymorphic. Cross‐sectional STEM provides evidence that 6 L thick In_2_O_3_ (Figure 2b) evolves from being epitaxial to SiC (first two layers) to a cubic structure. The interlayer distance is ≈2.5 Å, which aligns with the interlayer distance found in cubic In_2_O_3_ (001).^[^ ^17^ ^]^ This suggests that there is a special relationship of the first few layers to SiC, which is lost for thicker InO_x_. The preference for monolayer InO_2_ formation using a semi‐confined growth scheme with patterned graphene can be explained by charge neutrality and valence shell filling. Indium ions most commonly have 3^+^ formal charge, while oxygen ions without any covalent bond tend to have 2^−^ formal charge (e.g., bulk In_2_O_3_). Oxygen having one covalent bond tend to have 1^−^ formal charge, due to a similar full‐shell stability according to the octet rule (e.g., [OH]^−1^). In our system, oxygen has one covalent bond with Si in the SiC substrate and 1^−^ formal charge. Starting from the Si atom on the surface of the substrate, the formal charge on each atom is {Si^0^ : O^1−^ : In^3+^ : O^2−^ : In^3+^ : O^2−^ : In^3+^ : …}. Assuming all In and O atoms are fully commensurate with the substrate (equal number of atoms in each layer for SiC and InO_x_), the layering above the substrate can't extend indefinitely due to excess net charge. In fact, monolayer structure with charge balance {Si^0^ : O^1−^ : In^3+^ : O^2−^} and equal layer‐by‐layer stoichiometry, already creates a charge‐neutral and chemically‐stable structure where all shells are filled. This explains the observed detachment of extra layers in the multi‐layer structural relaxation and why, in bilayers that show no detachment, In atoms are ejected from the first layer to maintain charge neutrality. Hence, the thermodynamic ground state is reached when graphene is patterned, and In outward diffusion is allowed via a semi‐confined growth scheme, while InO_x_ is fully confined underneath a continuous graphene sheet, leading to the formation of multilayer (up to 6 L) InO_x_.

Although the structure in Figure 2a holds the basis for the monolayer InO_2_, the experimentally observed structures are more complex. First, DFT indicates that two nearly energetically degenerate structures exist with Si‐H‐C and Si‐C‐H stacking sequences (Table S1, Supporting Information), similar to monolayer GaO_2_.^[^ ^3^ ^]^ Both of these structures are experimentally observed in the cross‐sectional STEM images, where nanoscale variations in the atomic positions of In and top O atoms are evident in Figure 3a,b. This surface reversing is also observed in the surface oxides of III‐nitrides.^[^ ^18^ ^]^ In both ground state structures for monolayer InO_2_, the In and O atoms occupy distorted edge‐shared octahedral and corner‐shared tetrahedral positions, respectively, due to the polarity of the EG/SiC interface. Additionally, the distance between the top oxygen atoms in InO_2_ and graphene exhibits spatial variations ranging from 1.6 to 3 Å (Figure 3c), suggesting the formation of Gr─O covalent bonds, similar to C─O─Al bonding observed in graphene grown on sapphire.^[^ ^19^ ^]^ To investigate this, we created 44 crystal structures by modifying the lowest energy monolayer InO_2_ structure with Si‐H‐C stacking sequence (Figure 3a) and relaxed through ab initio DFT. We find that Gr─O bonding becomes energetically favorable in the presence of an In vacancy by 1.57 eV defect^−1^. Specifically, two to three Gr─O bonds are likely to form where there is one In vacancy, which is explained by charge difference plot. Additionally, clustering of In vacancies and Gr─O bonds (Figure 3d) is energetically more favorable than a sparse and uniform distribution of In vacancies by 0.47 eV defect^−1^ (Figure S11, Supporting Information). This likely results from regions with and without Gr─O bonds that favor shorter and larger interlayer distances between the oxygen and graphene layers, respectively, and clustering the Gr─O bonds reduces the energetic cost associated with these compromising interlayer distances. The simulated (Figure 3d) and experimental (Figure 3c) structures are compared by measuring the STEM intensity profiles along the *z*‐axis. The comparison in Figure 3e confirms that the distances between the top oxygen and In atoms, both with and without the Gr─O bond, match in the simulated and experimental structures. This supports that the simulated structure in Figure 3d is very close to the experimental product. The non‐van der Waals (vdW) interface between InO_2_ and graphene is also verified by our graphene exfoliation experiments, in which InO_2_ is peeled off along with graphene following nickel deposition and exfoliation using Scotch tape.^[^ ^20^ ^]^ The formation of Gr─O bonds due to In vacancies is also consistent with experimental observations. During high‐temperature oxidation of EG/In/SiC, indium deintercalation is favored, as evidenced by ReaxFF simulations, leading to the development of In_1−x_O_2_ regions. These regions, in turn, promote Gr─O bond formation to maintain charge balance. The structural differences between the graphene‐intercalated 2D InO_x_ structures in this study and those described in ref. [8], i.e., monolayer thickness and Gr─O bonding, are likely due to variations in the synthesis methods employed. Specifically, our study employs patterned graphene, in contrast to the continuous graphene used in ref. [8], which is associated with variations in InO_x_ thickness, typically resulting in the formation of multilayer InO_x_.

**Figure 4:**
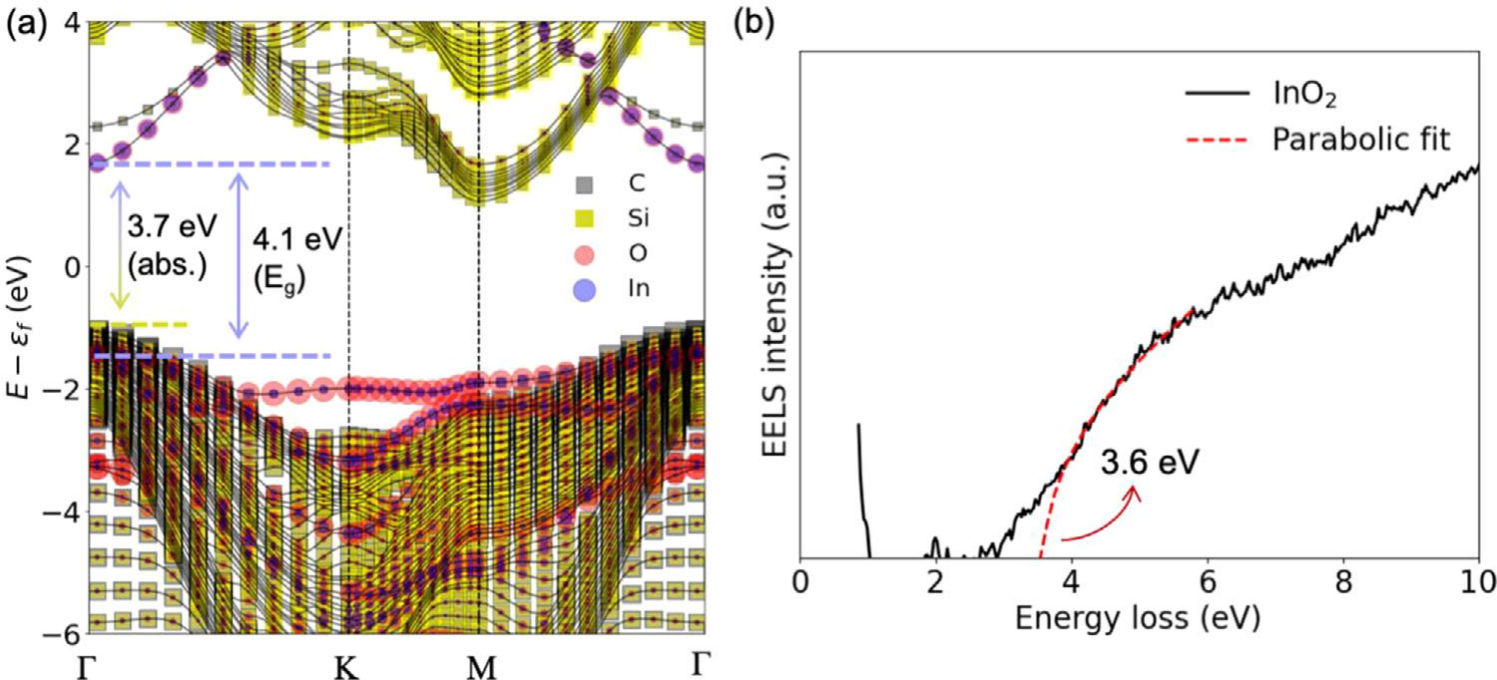
Calculated band structure of monolayer InO_2_ intercalated EG/SiC via DFT, demonstrating a direct bandgap of ≈4.1 eV for InO_2_ and 3.72 eV direct transition at Γ point. EELS measurement in TEM acquired from EG/InO_2_/SiC, demonstrating an absorption at ≈3.6 eV, matching with DFT results.

The monolayer InO_2_ exhibits a direct bandgap of ≈4.1 eV. Using the monolayer InO_2_ structure in Table S1 (Supporting Information) (Si‐H‐C stacking), the electronic band structure of the InO_2_/SiC heterostructure was calculated via DFT. Initially, we validated our DFT approach by calculating the band structure of pristine 4H‐SiC and 6H‐SiC, finding an indirect bandgap of 2.2 and 2 eV (Figure S12). This value is lower by ≈1 eV compared to established data for 4H and 6H‐SiC due to known underestimations in DFT calculations.^[^ ^21^ ^]^ To adjust for this, we added ≈1 eV to the calculated band structure results, leading to a calculated direct bandgap of ≈4.1 eV at the Γ point (**Figure** **4**a) for InO_2_, matching that of earlier theoretical calculations of monolayer InO,^[^ ^22^ ^]^ and larger than indirect bandgap observed in bulk In_2_O_3_ (≈2.7 eV).^[^ ^23^ ^]^

Intercalation of InO_2_ modifies the absorption characteristics of the heterostructure, while its emission properties remain similar to SiC. The lowest emission energy of the heterostructure matches that of SiC, as both the conduction band (CB) minimum and valence band (VB) maximum are still dominated by SiC states (Figure 4a). Additionally, there is an extra emission peak at 3.5 eV, which arises from transitions between CB minimum of SiC and VB maximum of InO_2_. Regarding absorption, prior to the intercalation, the optical gap (the energy gap at Δ*k* = 0) in 4H‐SiC is 4.4 eV at the 𝙼 point.^[^ ^24^ ^]^ With InO_2_ intercalation, new states at the CB emerge at the Γ point, reducing the optical gap of the heterostructure to 3.72 eV. Low‐loss EELS measurements, which correlate to the joint density of states (JDoS), were employed to confirm these findings (Figure 4b). This technique is particularly effective for measuring direct band transitions in the material as inelastic scattering events rapidly decrease with increasing momentum change, making indirect transitions with high momentum change less detectable.^[^ ^25^ ^]^ The background‐subtracted InO_2_ EELS spectrum was fitted with a parabolic curve (*E‐Eg*)^0.5^, reflecting the typical JDoS behavior for direct transitions near the band edge.^[^ ^26^ ^]^ The absorption, observed at 3.6 eV, aligns with the direct transition from SiC to InO_2_ bands at the Γ point, as predicted by the calculated band structure (Figure 4a). It's noted that the extracted absorption in EELS highly depends on the chosen energy window for the fit, as variations in this window yielded fit results ranging from 3.43 to 3.74 eV (Figure S13, Supporting Information).

The structural evolution of InO_2_ from 3D to 2D impacts its phonon band structure as well. Figure S14a (Supporting Information) demonstrates the Raman spectra taken from In, InO_2_ and mixed In/InO_2_ intercalated EG/SiC. Upon full oxidation, In ULF peaks (17, 45, and 96 cm^−1^) disappear and InO_2_ peaks are not detectable. However, in a partially oxidized sample, 2D InO_2_ peaks emerge at 295, 323, 421, and 450 cm^−1^, likely due to surface enhanced Raman scattering (SERS) effect when near metallic In. The origin of these peaks is attributed to encapsulated InO_2_, confirmed by DFT calculations that match with experimental peak positions (Figure S15, Supporting Information). Compared to the bulk In_2_O_3_ Raman peaks at 303, 360, and 492 cm^−1^,^[^ ^27^ ^]^ 2D InO_2_ peaks are red shifted which is attributed to the expansion of In─O bonds influenced by Si─O and C─O bonds present. Structural calculations show that the bond distance between the top oxygen and In increases from 2.05 to 2.2 Å due to Gr─O bonding (Figure S17, Supporting Information). Likewise, the presence of Si─O bonds increases the bottom O─In distance to 2.25 Å, larger than the one in bulk In_2_O_3_ (2.14 Å),^[^ ^28^ ^]^ explaining the observed red shift in the Raman spectra due to bond expansion at the EG/SiC interface.

**Figure 5:**
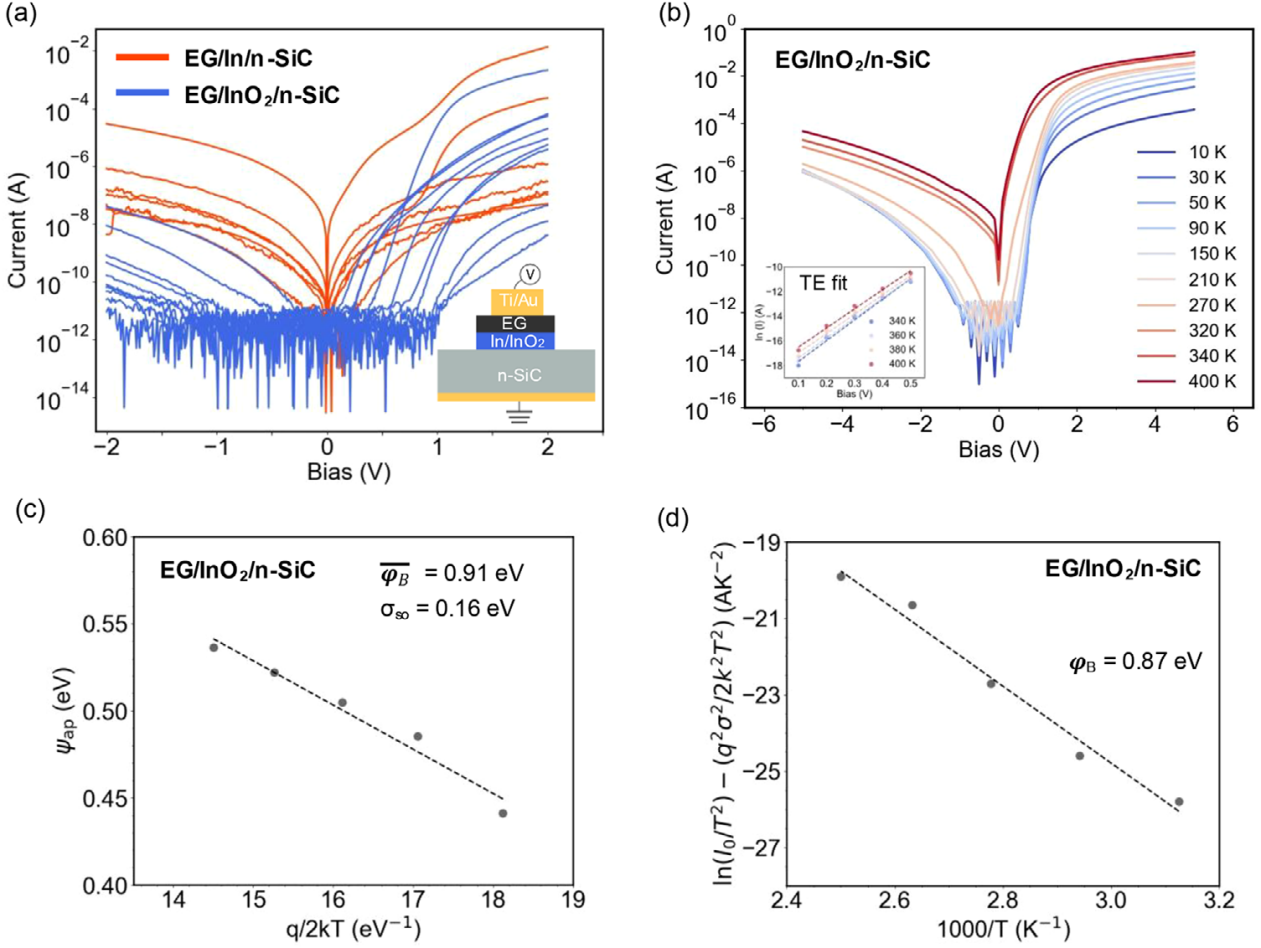
Current–voltage (*I*–*V*) curves taken from In and InO_2_ intercalated EG/n‐SiC vertical Schottky diodes, demonstrating reduced reverse leakage current with InO_2_ interfacial layer a). Schematic for the device structure is given in the inset. Temperature‐dependent (10–400 K) *I*–*V* taken from EG/InO_2_/n‐SiC diode with a forward bias thermionic emission fit given in the inset b). Apparent barrier height versus q/2kT c) and modified Richardson plot d) of the EG/InO_2_/n‐SiC diode according to the Gaussian distribution of the barrier heights.

Electronic transport properties of In intercalated EG/n‐SiC Schottky diodes were evaluated before and after oxidation, demonstrating a 10^5^‐fold enhancement in the rectification ratio as a result of the oxidation. As intercalation transforms EG/n‐SiC junction from ohmic to Schottky due to the elimination of buffer layer and E_F_ depinning,^[^ ^10^ ^]^ In intercalated EG/n‐SiC diode is also fabricated for comparison. As top and bottom electrodes, 5/40 nm thick Ti/Au is deposited on EG and n‐SiC, respectively. Device fabrication steps are shown in Figure S18 (Supporting Information). First, we assessed the contact properties of the EG/Ti/Au junction (top electrode) to Ohmic behavior and that the barrier observed in diode measurements is originating from the modulations at the EG/n‐SiC interface. Using the circular transfer length method (CTLM), the contact resistivity (*ρ_c_*) is extracted as 2.06 × 10^−6^ Ωcm^2^ (Figure S19, Supporting Information), which confirms a low resistance ohmic contact. For the bottom electrode, graphene grown on the C face of SiC facilitates ohmic contact formation with Ti/Au (Figure S20, Supporting Information).^[^ ^10^ ^]^ In and InO_2_ intercalated EG/n‐SiC diodes are evaluated by grounding the n‐SiC substrate (**Figure** **5**a). Schottky like behavior is verified for both samples, which is attributed to the E_F_ depinning due to Si─In (In) or Si─O (InO_2_) bond formation via intercalation, similar to H intercalated EG/n‐SiC diodes (Figure S21, Supporting Information).^[^ ^10^ ^]^ However, there is up to a 10^5^ × difference between the rectification ratio (RR) of In and InO_2_ intercalated devices with a max RR of ≈70 and ≈2 × 10^6^, respectively, indicating suppressed reverse leakage current with InO_2_ intercalation. We note that additional material development is needed for improved device reliability and uniformity. This is evident when comparing device‐to‐device performance: forward bias *V_th_* varies between 0.07 and 0.43 V for In (0.34 ∓ 0.19 V), and 0.21 and 1.33 V for InO_2_ (0.92 ∓ 0.47 V) devices. Similarly, RR is varied between 1.6 and 70 for In and 135 ‐ 2.18 × 10^6^ for InO_2_ devices. For In intercalated devices, this is likely to be a result of device fabrication on step edges, where no intercalant is found or on non‐intercalated terrace regions. On the other hand, the high leakage current observed under reverse bias with EG/InO_2_/n‐SiC diode (on the order of nA at –2 V) occurs sporadically due to device‐to‐device variability. This variability arises mainly from spatial discontinuities of InO_2_ on the nanometer scale (Figures S22 and S23, Supporting Information).

Temperature‐dependent electrical analysis on the EG/InO_2_/n‐SiC junction, along with nanoscale transport properties examined via C‐AFM, reveals nanoscale inhomogeneities in the barrier height. We first conducted temperature‐dependent *I*–*V* measurements (10 and 400 K) with EG/InO_2_/n‐SiC to extract the barrier height (Figure 5b). The current in reverse bias, remains constant between 10 and 150 K, indicating that tunneling is dominant < 150 K. The fit of the reverse bias current at 10 K with Fowler Nordheim Tunnelling (FNT) model exhibits a linear relationship (ln (*I/V^2^*) vs *1/V*), indicating tunneling as dominant conduction mechanism for low temperatures (Figure S21a, Supporting Information). In the forward bias, the *I*–*V* data is examined with the thermionic emission (TE) model (see methods).^[^ ^29^ ^]^ From the Richardson plot (Figure S21b, Supporting Information), the barrier height and Richardson constant is extracted as 0.19 eV and 139 Acm^−2^K^−2^, respectively. Although extracted A^*^ is close to the theoretical Richardson constant of 4H SiC (146 Acm^−2^K^−2^),^[^ ^30^ ^]^ the barrier height is lower than expected. As an alternative method, we extracted the barrier height from Equation 4 (see Experimental Section) by using theoretical A^*^ for 4H‐SiC for each temperature. As shown in Table S4 (Supporting Information), with higher temperatures, the barrier height increases from 0.39 to 0.49 eV and the ideality factor reduces from 3.87 to 1.76. Such a temperature‐dependent behavior of 𝜑_B_ and *n* is attributed to the inhomogeneity of the barrier height. At low temperature, electrons without sufficient energy can only surmount patches with lower Schottky barrier. As the temperature increases, more electrons gain sufficient energy to overcome higher barrier, leading a higher apparent Schottky barrier height. Based on Tung,^[^ ^31^ ^]^ a negative correlation between the apparent Schottky barrier and the ideality factor indicates the ideality factor decreases with increasing temperature. Lateral inhomogeneities at the junction have also been confirmed by conductive AFM (C‐AFM). The atomic‐resolution current map, shown in Figure S22a (Supporting Information) (10 nm × 10 nm), verifies the presence of high and low conductivity regions within the sample. Current–voltage curves acquired from different regions on the sample via C‐AFM demonstrate variations in *V_th_* in the reverse bias from –4 to –6 V (Figure S22b, Supporting Information), verifying the nanoscale inhomogeneities at the EG/InO_2_/n‐SiC junction. As shown in the post‐oxidation AES In KLL map (Figure 1h), nanoscale deintercalated regions exist, which contribute to the low φ_*B*_ regions as EG/n‐SiC junction is ohmic due E_F_ pinning by the Si dangling bonds.

Given these observations, vertical transport has been analyzed using single Gaussian distribution theory. The lateral inhomogeneity can be described by a Gaussian distribution with a mean barrier height ($\varphi_{B}$) and a standard deviation (*σ*).^[^ ^32^ ^]^ According to single Gaussian distribution theory, $\bar{\varphi_{B}}$ can be expressed by the following model:

(1) $$\varphi_{\mathrm{ap}}=\varphi_{B}-\frac{q\sigma^{2}}{2\mathrm{kT}}$$

where φ_*ap*_ and $\varphi_{B}$ are apparent and mean barrier heights, *σ* and is the standard deviation of the barrier height distribution which is a measure of the barrier height homogeneity. The experimental φ_*ap*_ versus *q/2kT* is shown in Figure 5c, where a linear relationship is seen. From the slope and the intercept of the φ_*ap*_ versus *q/2kT* plot, $\varphi_{B}$ and *σ* were extracted as 0.91 and 0.156 eV, respectively. Considering the lateral inhomogeneities in the barrier, a modified Richardson model can be expressed as:^[^ ^30^ ^]^

(2) $$\ln\frac{J_{0}}{T^{2}}-\left(\frac{q^{2}\sigma^{2}}{2k^{2}T^{2}}\right)=\ln\left(A^{*}\right)-\frac{q\varphi_{B}}{kT}$$

$\ln\frac{J_{0}}{T^{2}}-\left(\frac{q^{2}\sigma^{2}}{2k^{2}T^{2}}\right)$ versus *1/T* should give a straight line with the slope proportional to 𝜑_B_. Figure 5d presents the plot calculated with *σ* obtained from Figure 5c. Linear fit to the plot represents the true activation energy plot, where 𝜑_B_ from graphene to SiC is extracted as 0.87 eV. These electrical measurements with EG/InO_2_/n‐SiC verify Schottky barrier formation with InO_2_ intercalation and demonstrate the potential of this structure to be used as tunnel or filter barriers in 2D/3D hybrid hot electron transistors (HETs). When utilized as a tunnel barrier, the emitter current density reaches 10^5^ A cm^−^^2^, which is significantly higher than those used in graphene‐based HETs,^[^ ^33^, ^34^, ^35^ ^]^ attributed to the forward‐biased MOS‐based Schottky diode with a monolayer‐thick interfacial layer. On the other hand, when the EG/InO_2_/n‐SiC heterostructure serves as a filter barrier, it can suppress leakage current due to its high rectification ratio (RR). Unfortunately, device reliability remains a challenge, as evidenced by the large standard deviation in Vth of 0.47 V and RR of ≈10⁴. This is likely due to nanoscale non‐uniformities, such as variations in the EG‐InO_2_ interlayer distance, the presence of In vacancies, and deintercalated regions.

## 3 Conclusion

A new member of the 2D insulator family, monolayer InO_2_, synthesized by intercalating epitaxial graphene, is introduced. The influence of graphene patterning and graphene lateral size on the thickness and structural properties of 2D InO_x_ is shown, where monolayer and multilayer indium oxide is grown using patterned and continuous graphene, respectively. Extensive structural analysis via TEM and DFT indicates monolayer InO_x_ is generally formed at the EG/SiC interface and is explained based on the octet rule and charge neutrality principles. Additionally, DFT simulations confirmed that Gr─O bonding is stabilized by In vacancies, a consequence of In deintercalation during oxidation, highlighting the significant effect of the synthesis route on the structure of intercalated compounds. Moreover, monolayer InO_2_ exhibits a direct bandgap of ≈4.1 eV, ≈1.5× the indirect bandgap of bulk In_2_O_3_ (≈2.7 eV). Finally, Schottky diodes with InO_2_ and In intercalated EG/n‐SiC were fabricated, where a significant reduction in reverse leakage current with the InO_2_ intercalated device is demonstrated, achieving a maximum rectification ratio of 2 × 10^6^ (10^5^× higher than pre‐oxidation) with a barrier height of 0.87 eV. These findings illuminate several facets of materials science including control over intercalant thickness through graphene patterning, the synthesis‐structure‐property relationship, and vertical electronic transport in intercalated EG/n‐SiC structures.

## 4 Experimental Section

### Epitaxial Graphene Growth

n‐type 4H‐SiC wafers (Xiamen Powerway Advanced Material) were first cleaned with acetone, isopropyl alcohol (IPA), and nanostrip via ultrasonication. Then, to remove the surface oxide and polishing scratches from the SiC surface, the wafer was annealed in H_2_ at 1500 °C for 30 min (700 Torr, 10%/90% H_2_/Ar). Then, Si was sublimated from the SiC (0001) surface, and epitaxial graphene was formed at 1800 °C, 300 Torr for 30 min under Ar flow.

### Indium Oxide Intercalation

In intercalation was first performed using an STF‐1200 horizontal tube furnace fitted with a 1‐inch outer diameter quartz tube. An alumina crucible was used to hold 10 × 10 mm^2^ EG/SiC substrates, which were placed with EG on the Si face of SiC facing downward, toward the inside of the crucible. Then, ≈30 mg of In powder (Alfa Aesar, −325 mesh, 99.99%) was placed in the crucible directly beneath the EG/SiC substrate. The crucible with the sample and the respective metal precursor was then loaded into the tube furnace and evacuated to ≈3 mTorr. After pressurizing the system to 500 Torr with Ar, the furnace was heated to 800 °C with a ramp rate of 20° min^−1^ under an Ar flow of 50 sccm. Metal evaporation was carried out on EG at 800 °C, 500 Torr for 30 min, and then the furnace was cooled down to room‐temperature. Oxidation of In was conducted by annealing the as‐intercalated EG/In/SiC under O_2_/Ar (10/50 sccm) at 600 °C for 30 min in a rapid thermal annealing (RTA) furnace (OTF‐1200X‐4‐RTP).

### Raman Spectroscopy

Raman Spectroscopy was performed with a Horiba LabRam Raman system using a laser with 532 nm wavelength and power of ≈4 mW. An ultra‐low frequency filter was used to filter out the Rayleigh signal. Double sweep spectra were taken with an accumulation time of 30 s total using a grating with 300 grooves mm^−1^. Raman mapping was done using the SWIFT ultra‐fast imaging technique with varying pixel resolution (typically 0.8 × 0.8 µm). A Si wafer was used for calibration.

### XPS

The measurements were carried out with a Physical Electronics Versa Probe III equipped with a monochromatic Al Kα X‐ray source (hν = 1486.7 eV) and a concentric hemispherical analyzer. High‐resolution spectra were obtained over an analysis area of 200 × 200 µm^2^ (30 × 30 µm^2^ for patterned samples) with a pass energy of 29 eV for C 1s and In 3d regions. O 1s and Si 2p regions were acquired with a pass energy of 55 eV. As the samples were electrically contacted to the XPS stage, energy calibration was not done. In this case, Fermi level of the XPS spectrometer aligns with that of sample. This was confirmed by checking the peak positions in the spectra taken with neutralizer on and off.

### AES

AES maps were acquired with Physical Electronics Versa Probe III equipped with Auger electron spectroscopy using an electron beam with an energy of 10 keV and a current of 5 nA. For AES mapping, a three‐point acquisition method was used for the intensity calculation at each pixel, where a single point was used to define the peak intensity, and two points were chosen to define the background intensity. Maps were the average of 15 and 5 frames for In and O, respectively.

### AFM

The topography images were acquired with a commercial instrument (Bruker, Dimension Icon) in the PeakForce tapping mode by using a ScanAsyst‐Air probe with a constant force of 1 nN.

### C‐AFM

The C‐AFM experiments were performed under uncontrolled ambient conditions (temperatures of 30–35 °C, measured near the sample, and relative humidity levels of 30–40%), using a commercial instrument (Asylum Research, Cypher VRS). The experiments utilized a diamond‐coated, conductive AFM probe (Nanosensors, CDT‐NCHR) with a normal spring constant of 58 N m^−1^. The C‐AFM image in Figure S22a (Supporting Information) (10 × 10 nm^2^) was recorded with a high scan frequency of 15.62 Hz, under the application of a low bias voltage of 5 mV, conditions conducive to atomic‐resolution imaging under ambient conditions.^[^ ^36^ ^]^ The interaction force between the tip and the sample was kept at the snap‐in level, with no additional application of normal force. Current–voltage curves (Figure S22b, Supporting Information) were acquired with a commercial instrument (Bruker, Dimension Icon) using an SCM‐PIT‐V2 probe.

### STEM, EDS, and EELS

For the STEM images shown in Figure 2a,d,e,f, the cross‐sectional samples were prepared using a FEI Helios 660 focused ion beam (FIB) system. A thick protective amorphous carbon layer was deposited over the region of interest then Ga+ ions (30 kV then stepped down to 1 kV to avoid ion beam damage to the sample surface) were used in the FIB to make the samples electron transparent for STEM images. High‐resolution STEM was performed at 300 kV on a dual spherical aberration‐corrected FEI Titan G2 60‐300 S/TEM. All the STEM images were collected by using a high‐angle annular dark field (HAADF) detector with a collection angle of 50–100 mrad. EDS elemental maps of the sample surface were collected by using a SuperX EDS system under STEM mode, which has four detectors surrounding the sample. The cross‐sectional samples in STEM images in Figure 2b, Figures S2, and S3 (Supporting Information) were prepared using a Focused Ion Beam Scanning Electron Microscope (FIB‐SEM) equipped with a Ga ion gun (Thermo Fisher Helios 5). The initial tungsten (W) deposition was performed using electron beam deposition to form a 100 nm protective layer on the surface. This was followed by ion beam deposition of a thicker W film (5–6 µm) over the region of interest to protect the surface from ion damage during FIB milling. STEM micrographs were captured, and elemental maps were obtained through STEM‐EDS using a double‐corrected HRTEM/STEM (TFS Spectra Ultra, operating at 300 keV) and an Ultra‐X EDS detector. The EDS data was processed using Velox 3.0 EDS Software.

For absorption analysis on InO_2_ intercalated EG/SiC a line scan using EELS under STEM mode was performed across the region of interest. A GIF Quantum 963 system was used to collect all the EELS data. By using a X‐FEG high brightness electron gun with a monochromator, the energy resolution is ≈0.1 eV. EELS spectra were collected using a C2 aperture size of 70 µm, camera length of 38 mm, entrance aperture of 2.5 mm, and a dispersion of 0.1 eV pixel^−1^. This corresponds to convergence and collection semi‐angles of 9.3–18.7 mrad, respectively. Ideally, graphene should be removed from the InO_2_ surface to avoid interference in the EELS spectrum from graphene's plasmonic contributions. However, attempts to remove graphene resulted in the detachment of InO_2_ as well because of covalent bonding between graphene and oxygen at the interface. Consequently, EELS measurements were performed with graphene intact. Initially, the zero‐loss peak was subtracted using a power law fit, and then the spectrum from unintercalated graphene was subtracted from that of InO_2_.

### Device Fabrication

The Schottky diodes were fabricated via optical and e‐beam lithography. The steps before InO_2_ formation, i.e., alignment mark and graphene etching, were made with optical lithography, while the steps after were made with e‐beam lithography. Device fabrication steps are shown in Figure S18 (Supporting Information). First, an alignment mark layer was made by etching n‐SiC ≈500 nm deep with SF_6_ plasma. Then, circular graphene patterns with a diameter ranging from 3 to 30 µm were etched with N_2_ plasma in 15 s. Following the InO_2_ formation, graphene was contacted via Ti/Au (5/40 nm) lift‐off in a circular shape with 1 µm diameter. Note that the device active area was ≈0.28 µm^2^ as the Al_2_O_3_ etch window was circular with 0.6 µm diameter. The recipes used for the device fabrication are given in Table S3 (Supporting Information). To measure the contact resistance of graphene/Ti/Au, EG was grown on insulating 6H‐SiC (0001). The details of the contact resistance measurements and calculations are given in Figure S19 (Supporting Information). After contact lift‐off, graphene was etched by N_2_ plasma outside of the active area to minimize vertical transport through non‐intercalated graphene patches. In this layer, no lithography was used as metal contact protects the underlying InO_2_/EG from plasma damage. Since the as‐grown EG/n‐SiC junction was ohmic, this etching step was crucial to minimize regions with low Schottky barrier height (SBH) and obtain a higher rectification ratio (RR). Before depositing the Al_2_O_3_ isolation layer, a seed layer of Al (2 nm) was deposited on the sample using e‐beam evaporation (0.1 Å/sec). Then, 30 nm Al_2_O_3_ was deposited via ALD at 150 °C using trimethyl aluminum and water. This layer was to prevent leak from the metal leads to the n‐SiC substrate. To open the Ti/Au contacts, Al_2_O_3_ was etched using BCl_3_/Cl_2_ plasma for 60 s via e‐beam lithography, corresponding to 35 nm Al_2_O_3_. Over‐etching was employed to make sure all the Al_2_O_3_ is cleaned. Since Au was a good etch stop for BCl_3_ and Cl_2_ plasma, the sample easily survives with longer etching. For the metal leads and pads, Ti/Au (10/120 nm) lift‐off was employed using e‐beam lithography.

The forward‐bias Vth for each diode was determined using a consistent reference current of 0.1 nA from the measured current–voltage curves.

### Models for Electrical Transport Analysis

In the forward bias, since current increases with temperature, the *I*–*V* data was examined with the simplified thermionic emission (TE) model,^[^ ^29^ ^]^ as described below:

(3) $$J=J_{0}\times \exp\left(\frac{qV}{nkT}\right)$$

(4) $$J_{0}=A^{*}T^{2}\times \exp\left(\frac{-q\varphi_{B}}{kT}\right)$$

where *J* is the forward bias current density, *J_0_* is the saturation current density, *V* is the applied bias, *n* is ideality factor, *k* is Boltzmann constant, *T* is temperature, A^*^ is the Richardson constant of 4H‐SiC, and 𝜑_B_ is the barrier height from EG to SiC. Equation (3) can be rewritten as:

(5) $$\ln\left(J\right)=\ln\left(J_{0}\right)+\frac{qV}{nkT}$$

where *ln (J)* versus *V* plot should yield a linear relationship if thermionic emission was the dominant mechanism in the forward bias. At low bias (<0.5 V), *ln (J)* versus *V* plot yields a straight line (Figure 4b inset) for T > 320 K. Note that linearity in *ln (J)* versus *V* is lost for V > 0.5 V, which is attributed to the activation of FNT at higher bias. To extract the barrier height, 𝜑_B_, from forward bias characteristics, saturation current density, *J_0_*, was extracted at temperatures 320–400 K from the *y*‐axis intercept of the ln *(J)* versus *V* plot. Then, using the Equation (6), which is modified from Equation (4), barrier height (𝜑_B_) and Richardson constant (A*) are extracted from the slope and intercept of $\ln\frac{J_{0}}{T^{2}}\;$ versus *1/T* plot, as 0.19 eV and 139 Acm^−2^K^−2^, respectively (Figure S21b, Supporting Information).

(6) $$\ln\frac{J_{0}}{T^{2}}=A^{*}\times \frac{-q\varphi_{B}}{kT}$$

### DFT

Structural stability calculations for InO_x_ were done by ab‐initio DFT implemented by software package, VASP. Projected Augmented Wave method with PBE functional and a plane wave basis set were used. Gamma centered k‐mesh were used with step size ≈2Pi/24 A^−1^ along the two dimensions of the slabs. All structures were relaxed with fixed unit cell whose lattice parameters along the two relevant dimensions are set to be those of [0001] of 4H‐SiC.

Metal binding energy calculations with different thickness (Figure S9, Supporting Information) were conducted using Quantum Espresso. All structural relaxations in this work were performed with pseudopotentials based on the Projector Augmented Wave method. The Perdew–Burke–Ernzerhof (PBE) form of the Generalized Gradient Approximation (GGA) was employed to model the exchange‐correlation functional. A 4  ×  4  ×  1 k‐point mesh within a Gamma‐centered Monkhorst–Pack scheme was applied for Brillouin Zone integration, with kinetic energy and density cutoffs set at 60 and 600 Ry, respectively, for a supercell with dimensions of 9.23  ×  9.23 Å^2^. The Marzari–Vanderbilt cold smearing scheme was used with a broadening parameter of 0.01 Ry. During geometry optimizations, the system was fully relaxed using the Broyden–Fletcher–Goldfarb–Shanno (BFGS) algorithm, with a total energy convergence threshold of 0.0001 Ry and a force threshold of 0.001 Ry Å^−1^. van der Waals interactions were accounted for using Grimme's semiempirical DFT‐D3 correction (zero damping). A vacuum layer of 20 Å was introduced perpendicular to the graphene sheets to minimize spurious interactions due to periodic boundary conditions. In the calculations, Si‐terminated 6H‐SiC (0001) was used, with the bottom layer saturated with hydrogen to reduce computational cost. Gallium atoms were deposited on the top sites of the surface Si atoms, with the second and third layers following an ABC stacking sequence starting from the substrate surface.^[^ ^1^ ^]^

For the calculated phonon band structure first‐principles calculations based on DFT^[^ ^37^ ^]^ were performed using the GPAW code,^[^ ^38^, ^39^ ^]^ which employs the projector‐augmented wave (PAW) method for electron–ion interactions.^[^ ^40^ ^]^ The Kohn–Sham potentials were evaluated on a real‐space grid with a spacing of 0.175 °A. The calculations used the linear combination of atomic orbitals (LCAO) mode, expanding Kohn–Sham wavefunctions in the double zeta polarized (dzp) basis set,^[^ ^41^ ^]^ which reduces computational cost due to its compact size. For the exchange‐correlation functional, the Generalized Gradient Approximation (GGA) in the PBE formulation was adopted.^[^ ^42^ ^]^ The structure was modeled as a slab with a vacuum region of 15 °A in the out‐of‐plane direction to minimize interactions between periodic images. A Monkhorst–Pack 3 × 3 × 1 k‐point grid was used for structural relaxation until residual atomic forces were below 1 meV °A^−1^.^[^ ^43^ ^]^ The optimized lattice parameters were 9.13 and 9.14 °A for the a and b lattice constants, respectively. Phonon frequencies and eigenvectors were computed within the finite displacement method (FDM) in the harmonic approximation using Phonopy,^[^ ^44^ ^]^ interfaced with ASE.^[^ ^45^ ^]^ A default displacement magnitude of 0.01 °A was used without additional supercell geometry for phonon and Raman calculations. Since DFT operates at zero temperature, temperature effects were not considered. Resonant Raman spectra were computed using the in‐house code^[^ ^46^ ^]^ implemented for GPAW version 22.1, based on third‐order perturbation theory.^[^ ^47^ ^]^ The code requires GPAW to operate in LCAO mode, and post‐processes the electron–phonon, and momentum matrix elements into Raman tensors in the finite difference approach. While the implementation relies on GPAW version 22.1, GPAW has since introduced an official Raman code in version 23.6.0.

### ReaxFF Molecular Dynamics Simulations

Since both In and Ga belongs to the same group in the periodic table and were chemical analogs of each other, a ReaxFF reactive force field developed by Niefind et al.^[^ ^48^ ^]^ for Graphene/Ga/O/SiC interactions was utilized in large scale molecular dynamics simulations. Each model was placed in a box with the cell dimensions of 19.3 nm × 13.7 nm × 1000 nm and each model consists of a Si‐terminated 6H‐SiC (0001) substrate, a bilayer graphene layer, and Ga layers ranging from 1 to 3 layers. Upon minimization, each model was first kept in an isothermal‐isobaric ensemble (NPT) at 300 K to relieve any artificial strain resulting from lattice mismatches at the interface of SiC/Ga/graphene, then subjected to annealing at a target temperature in a constant temperature and constant volume ensemble (NVT) with a temperature damping constant of 100 fs either without or under the O_2_ exposure. The details of temperature and time durations are given in the captions of the figures (Supporting Information). The time step was set to 0.1 fs. To control the temperature fluctuations, Berendsen thermostat was deployed. Newton equations of motion was integrated using Velocity verlet algorithm. VESTA was utilized for atomic illustration purposes. For the SiC/graphene model where the first layer of bilayer graphene is rotated by 30° with respect to the SiC substrate with the ten layers to reduce the lattice mismatch between the SiC and graphene to 0.2%. Additionally, to observe the graphene edge effect on the Ga de‐intercalation, thus mimicking the experimental setup, the periodicity of bilayer graphene was removed in all three directions

## Conflict of Interest

The authors declare no conflict of interest.

## Supporting information



Supporting Information

## Data Availability

The data that support the findings of this study are available from the corresponding author upon reasonable request.
