# Supplementary material for: 2D Indium Oxide at the Epitaxial Graphene/SIC Interface: Synthesis, Structure, Properties, and Devices
Source: Adv Mater. 2025 Nov 10;38(6):e16133. doi: 10.1002/adma.202516133 (PMC12848651; doi:10.1002/adma.202516133)
Supplement: Supplementary file 1 — Supporting Information [file ADMA-38-e16133-s001.docx]

**SUPPLEMENTARY INFORMATION**

**Two-dimensional Indium Oxide at the Epitaxial Graphene/SiC Interface: Synthesis, Structure, Properties, and Devices**

*Furkan Turker, Bohan Xu, Chengye Dong, Michael Labella III, Nadire Nayir, Natalya Sheremetyeva*, *Zachary J. Trdinich, Duanchen Zhang, Gokay Adabasi, Bita Pourbahari, Li-Syuan Lu, Wesley E. Auker, Ke Wang, Mehmet Baykara, Vincent Meunier, Nabil Bassim, Adri C.T. van Duin, Vincent H. Crespi, Joshua A. Robinson**

F. Turker, Z. J. Trdinich, D. Zhang, Li-Syuan Lu, V. Meunier, Adri C.T. van Duin, V. H. Crespi, J. A. Robinson

Department of Materials Science and Engineering

The Pennsylvania State University, University Park, PA, 16802, USA.

*Corresponding author’s email: jrobinson@psu.edu

F. Turker, C. Dong, V. H. Crespi, J. A. Robinson

Center for 2-Dimensional and Layered Materials

The Pennsylvania State University, University Park, PA, 16802, USA.

B. Xu, V. Meunier, V. H. Crespi, J. A. Robinson

Department of Physics

The Pennsylvania State University, University Park, PA, 16802, USA.

C. Dong, V. H. Crespi, J. A. Robinson

Two-Dimensional Crystal Consortium

The Pennsylvania State University, University Park, PA 16802, USA.

M. Labella III, Wesley Auker, Ke Wang

Materials Research Institute

The Pennsylvania State University, University Park, PA, 16802, USA.

N. Nayir

(1) Paul-Drude-Institute for Solid State Electronics

Leibniz Institute within Forschungsverbund Berlin eV., Hausvogteiplatz 5-7, 10117 Berlin, Germany

(2) Department of Physics Engineering

Istanbul Technical University, Maslak, Istanbul 34469, Turkey

N.Nayir, Adri C.T. van Duin

Department of Mechanical Engineering

The Pennsylvania State University, University Park, PA 16802, USA.

G. Adabasi, M. Baykara

Department of Mechanical Engineering

University of California Merced, Merced, California 95343, USA.

B. Pourbahari, N. Bassim

Department of Materials Science and Engineering

McMaster University, Hamilton, Ontario, L8S 4L8, Canada.

B. Pourbahari, N. Bassim

Canadian Centre for Electron Microscopy

Hamilton, Ontario, L8S 4L8, Canada.

V. H. Crespi, Adri C.T. van Duin, J. A. Robinson

Department of Chemistry

The Pennsylvania State University, University Park, PA, 16802, USA.

Natalya Sheremetyeva, V. Meunier, Adri C.T. van Duin, J.A. Robinson

Department of Engineering Science and Mechanics

The Pennsylvania State University, University Park, PA, 16802, USA.

**Keywords:** 2D indium oxide, intercalation, graphene, heterostructure, vertical Schottky diode

# Indium Intercalation

## Indium Intercalation with Continuous Graphene

As epitaxial graphene is high quality, low power O_2_ plasma treatment is applied to generate defects in graphene (1x1 cm^2^) prior to intercalation. Then, indium is intercalated by evaporating metallic indium precursor at 800 ^o^C, 500 Torr, under 50 sccm Ar flow in 30 minutes. High resolution X-Ray Photoelectron Spectra are given in Figure S1. SiC peak shift from 283.7 eV to 282.7 eV and the elimination of buffer layer peaks in C 1s show successful indium intercalation.^[1]^ Minimal oxygen peak in O1s, as well as Indium peak position at 443.8 eV verify that graphene protects intercalated indium from oxidation, similar to the Ref.^[2]^


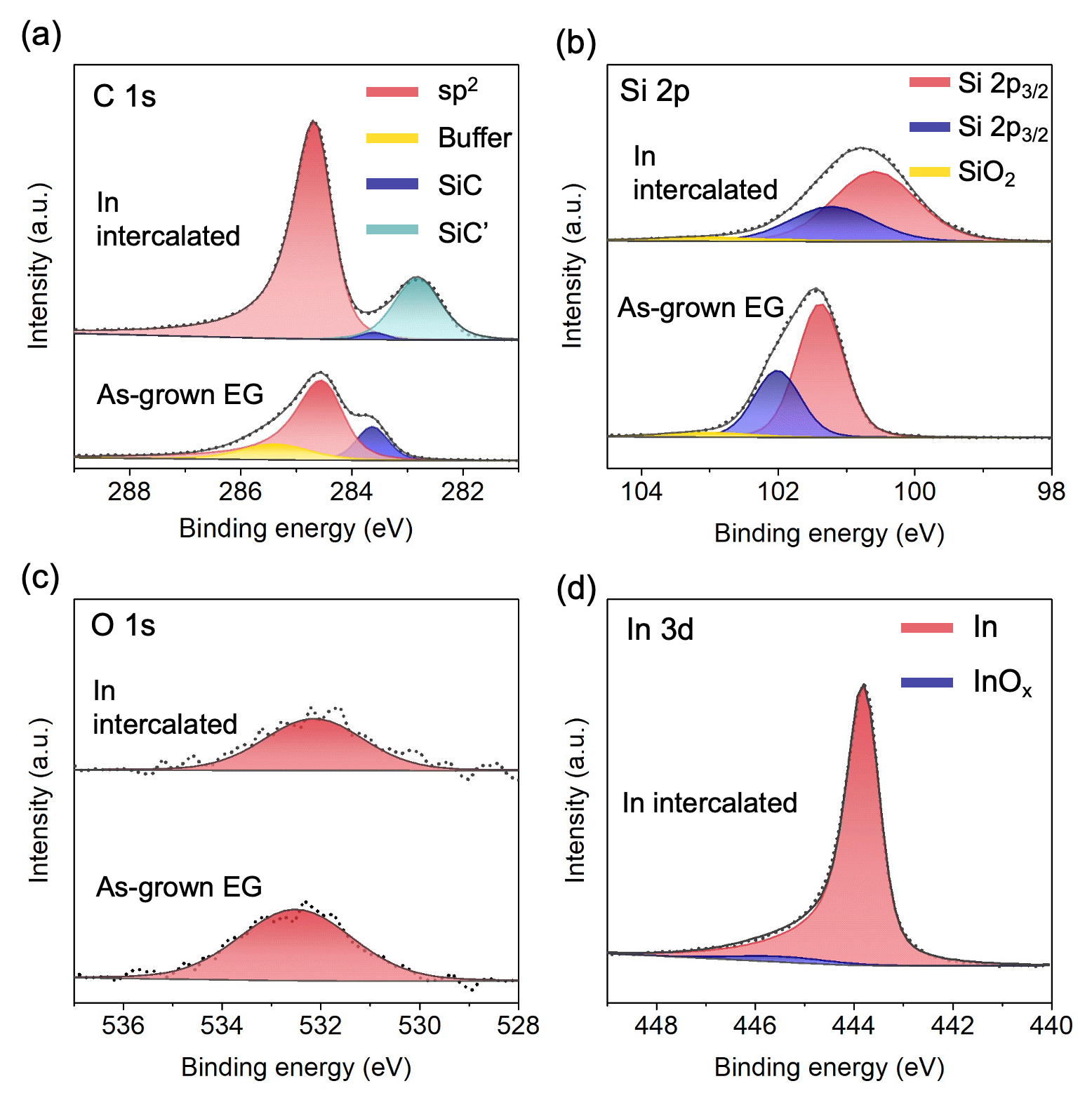


**Figure S1:** High resolution X-ray photoelectron spectra of the continuous EG/SiC sample before and after indium intercalation showing (a) C 1s, (b) Si 2p, (c) O 1s, (d) In 3d regions.

## Indium Intercalation with Patterned Graphene

In the 2^nd^ approach, epitaxial graphene is first patterned by optical lithography and then etched with O_2_ plasma to create circular graphene with 20 μm diameter. Then, EG/SiC is intercalated with indium via same method as the 1^st^ approach (see methods). Scanning electron microscope (SEM) image of the intercalated patterned graphene is given in Figure S2a. Raman spectrum taken from the circles presents metallic indium peaks in low frequency region (17, 45, 96 cm^-1^).^[3]^ Uniformity of the indium intercalation is verified by Raman mapping (Figure S2c, d), scanned for graphene G band at ~1600 cm^-1^ and indium peak at ~17 cm^-1^. Direct evidence for the bilayer indium intercalation is given in the cross-sectional STEM image, along with EDS elemental maps (Figure S2e).


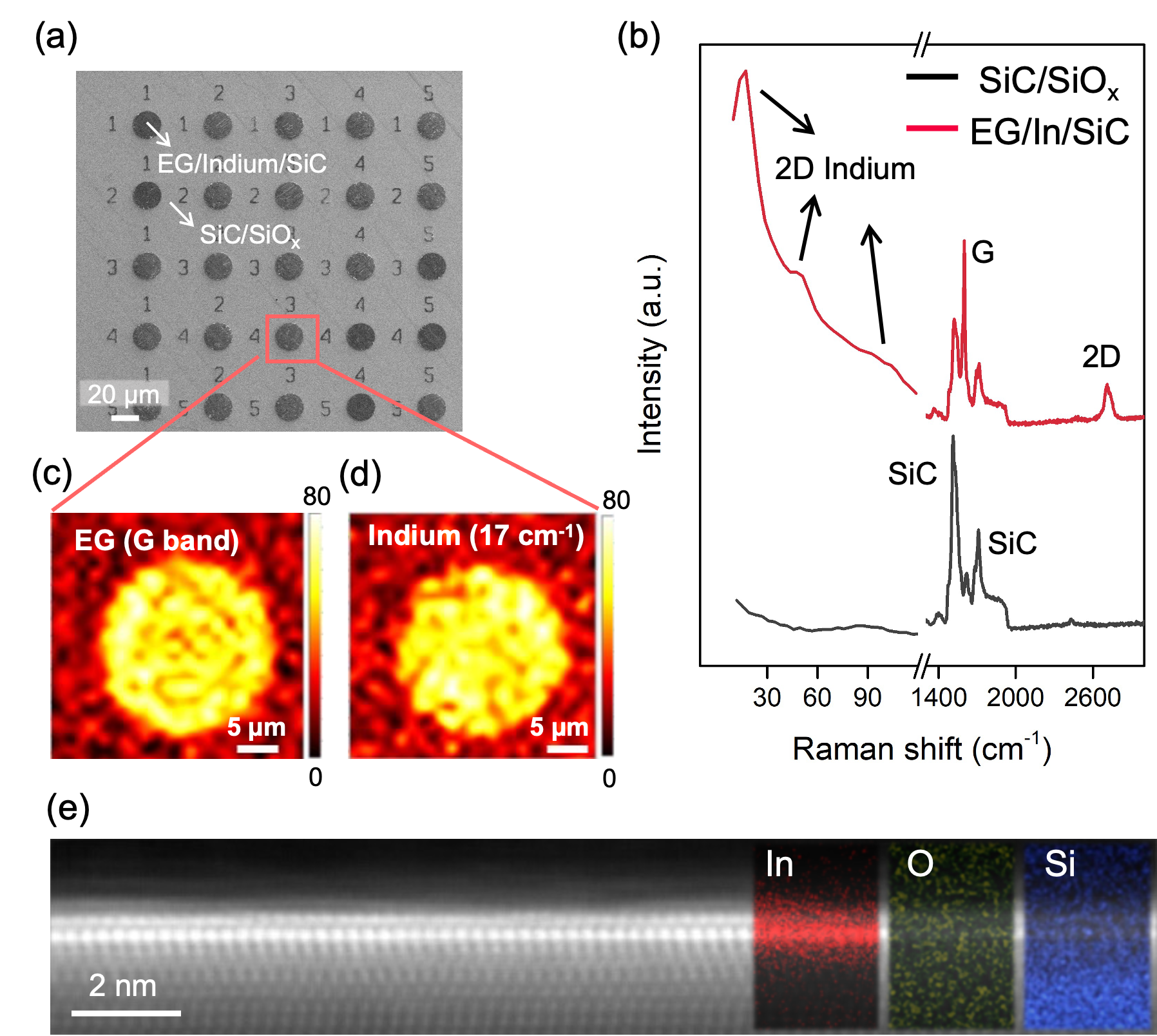


**Figure S2:** Raman, SEM, and STEM characterization of the patterned sample after indium intercalation. SEM image (a) shows that circular graphene patterns with 20 µm diameter are formed. Raman spectrum taken from within the circle exhibits metallic indium ultra-low frequency peaks at 17, 45, 96 cm^-1^. Uniformity of the intercalation is confirmed by Raman mapping where graphene G band at 1600 cm^-1^ (c) and metallic indium peak at 17 cm^-1^ (d) is continuous within the circle. Direct evidence for the intercalation is given in cross-sectional STEM image along with EDS elemental maps (e), showing epitaxial, bilayer indium at the graphene/SiC interface.

Low power O_2_ plasma treatment for graphene defect generation is not necessary for successful intercalation using the patterned graphene sample. Following graphene etching during lithography, the edges of the patterns remain defective (Figure S3a) as graphene D peak intensity increases toward the edge of the sample. This allows large area indium intercalation while keeping the graphene pristine. Cross-sectional STEM analysis verifies bilayer indium intercalation between EG and SiC and the absence of indium outside of the patterned region, where graphene is not present. (Figure S3b, c).


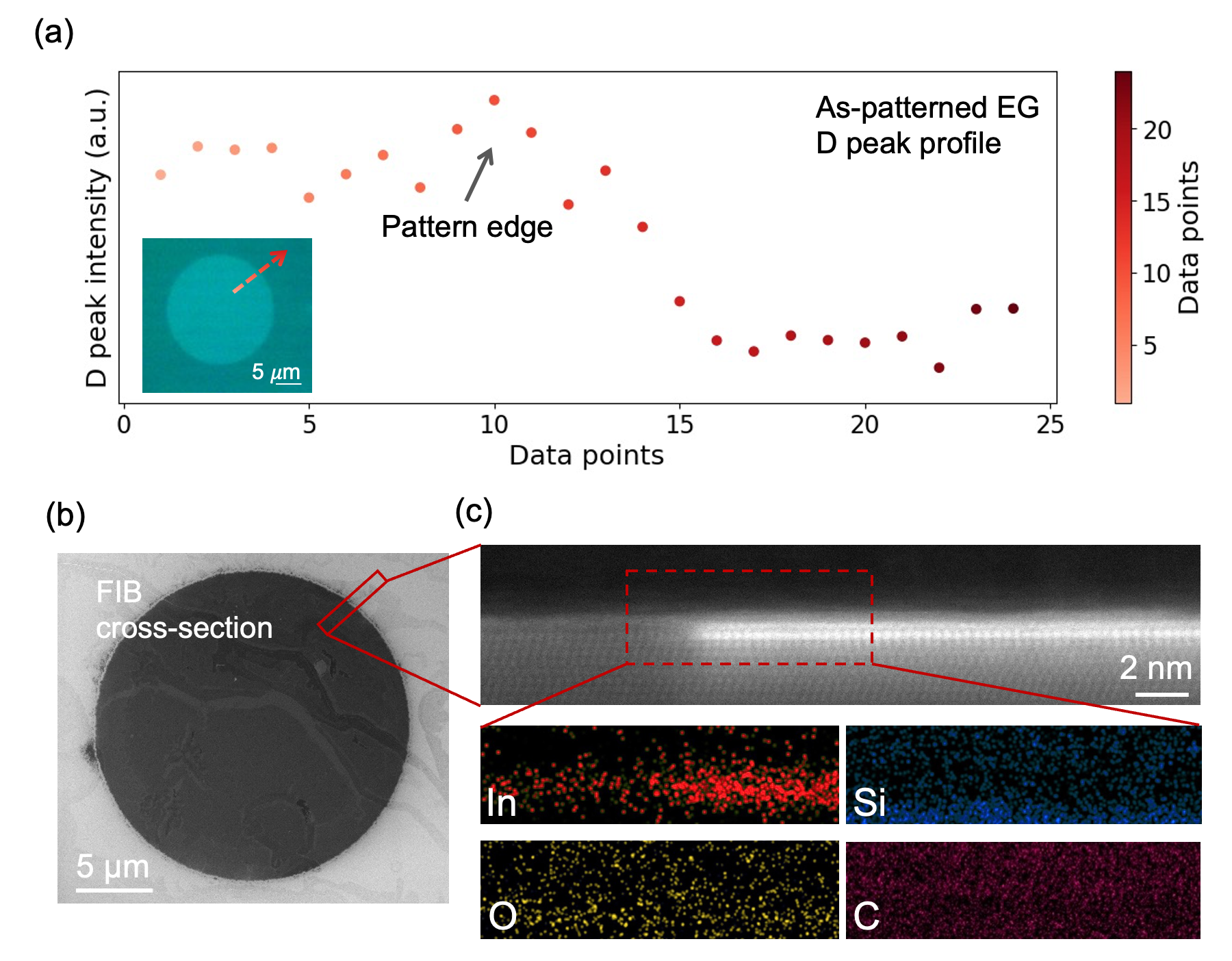


**Figure S3:** D peak intensity of patterned graphene from the center towards the edge before intercalation (a), demonstrating higher defect density at the edge of the pattern due to plasma etching during lithography. SEM image and cross-sectional STEM image along with EDS elemental maps of the patterned sample after indium intercalation, showing that 2D indium film forms underneath graphene.

Following the indium intercalation through patterned EG/SiC, bright features appear in optical micrographs (Figure S4b). Based on Auger Spectroscopy and the observation of graphene wrinkles in AFM near the particle (not shown here), these regions correspond to thick indium particles beneath graphene, which serve as indium sinks during intercalation. During oxidation, graphene initially cracks in these regions, presumably due to high stress (Figure S4d), leading to indium deintercalation. Importantly, the density of these particles reduces by increasing the graphene pattern size and diminish when the intercalation is conducted using the whole sample (1x1 cm^2^). To reduce the density of these indium particles, graphene is first patterned into large squares (~1.5 mm) and then further etched in the form of circles with 20 µm diameter via another lithography step (2 step etching via lithography). Note that indium intercalation is more successful when graphene is etched via lithography even if the graphene size is 1.5 mm X 1.5 mm than using the whole sample (1 cm X 1 cm), demonstrating indium can diffuse long distances through defective graphene edges (Figure S5). Optical micrograph and Raman indium peak mapping after 2^nd^ etching step are given in Figure S4f, g, verifying smoother surface and uniform intercalation. Importantly, graphene crack formation and indium deintercalation during oxidation are significantly suppressed via this method as SEM image presents smoother surface (Figure S4h).


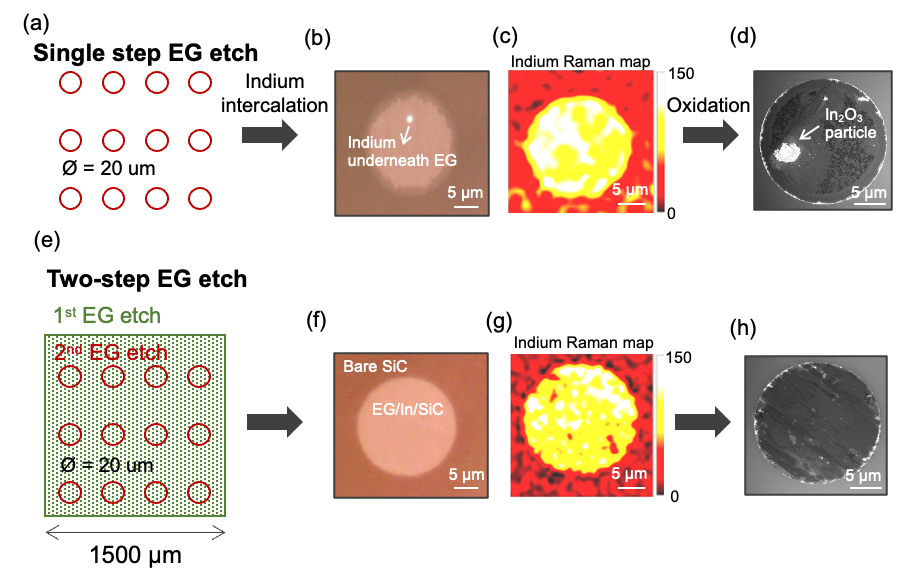


**Figure S4:** Comparison of single and two step etching of graphene for the 2D InO_x_ formation. Schematics showing the single step (a) and two-step (e) etching of EG where graphene is etched outside of circular patterns. In (e), first, graphene is patterned with 1.5 mm X 1.5 mm size for the indium intercalation and then etched again in the form of circles for the subsequent oxidation. Optical micrographs (b, f) and metallic indium Raman (17 cm^-1^) maps (c, g) taken from In/EG, showing that particle formation beneath EG can be avoided via 2 step etching. SEM images of the oxidized samples for single step (d) and two-step graphene etching (h), showing that graphene crack due to indium particle oxidation underneath EG is avoided via two-step etching.


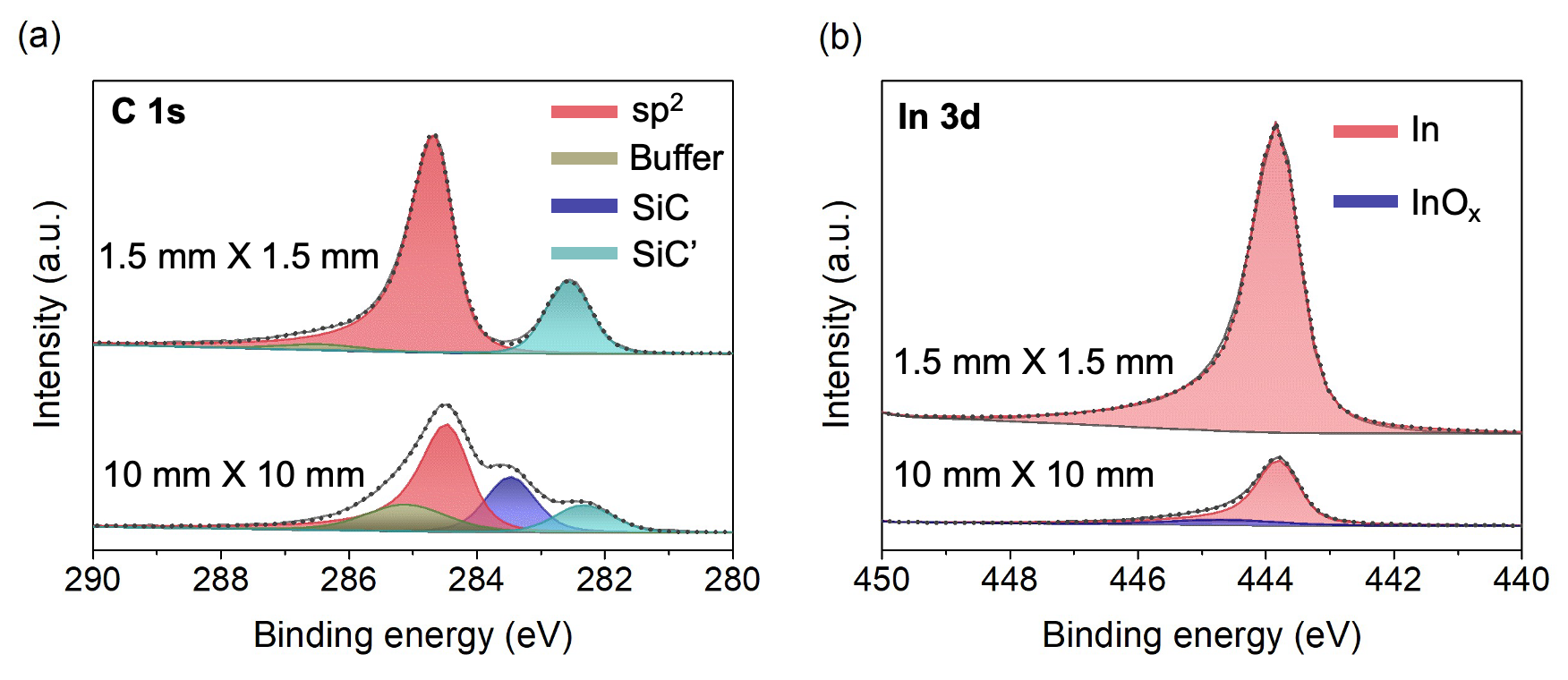


**Figure S5:** XPS high resolution C 1s (a) and In 3d (b) spectra taken from indium intercalated samples with sizes 1.5 mm X 1.5 mm (lithographically etched) and 10 mm X 10 mm (whole sample). SiC peak in C 1s spectra shifts to lower binding energy following indium intercalation (SiC’ peak) due to change in band bending with patterned sample (1.5 mm X 1.5 mm). Partial intercalation with 10 mm X 10 mm size sample is verified by the remaining unshifted SiC peak in C 1s spectrum along with lower intensity indium peak in In 3d region.

# InO_x_ Formation at the EG/SiC Interface

Oxidation of indium intercalated continuous and patterned graphene yields partial and fully oxidized indium, respectively. Partial oxidation of indium in continuous graphene sample is verified by Auger spectra taken from the bright/dark contrast in the optical micrograph (a). Indium MNN Auger peaks in bright/dark regions at ~403/400 eV correspond to metallic/oxide indium peak positions, respectively. Additionally, O KLL peak intensity in bright region is minimal (Figure S6c) compared to dark contrast and Raman spectrum taken from the bright region still presents metallic indium Raman peak at 17 cm^-1^. On the other hand, In MNN peak at 399.8 eV and the absence of metallic indium Raman peak at 17 cm^-1^ from the patterned sample verify successful indium oxidation. Uniformity of indium and oxygen within the circle is presented in Auger In MNN and O KLL maps in Figure 1h, j in the main text.


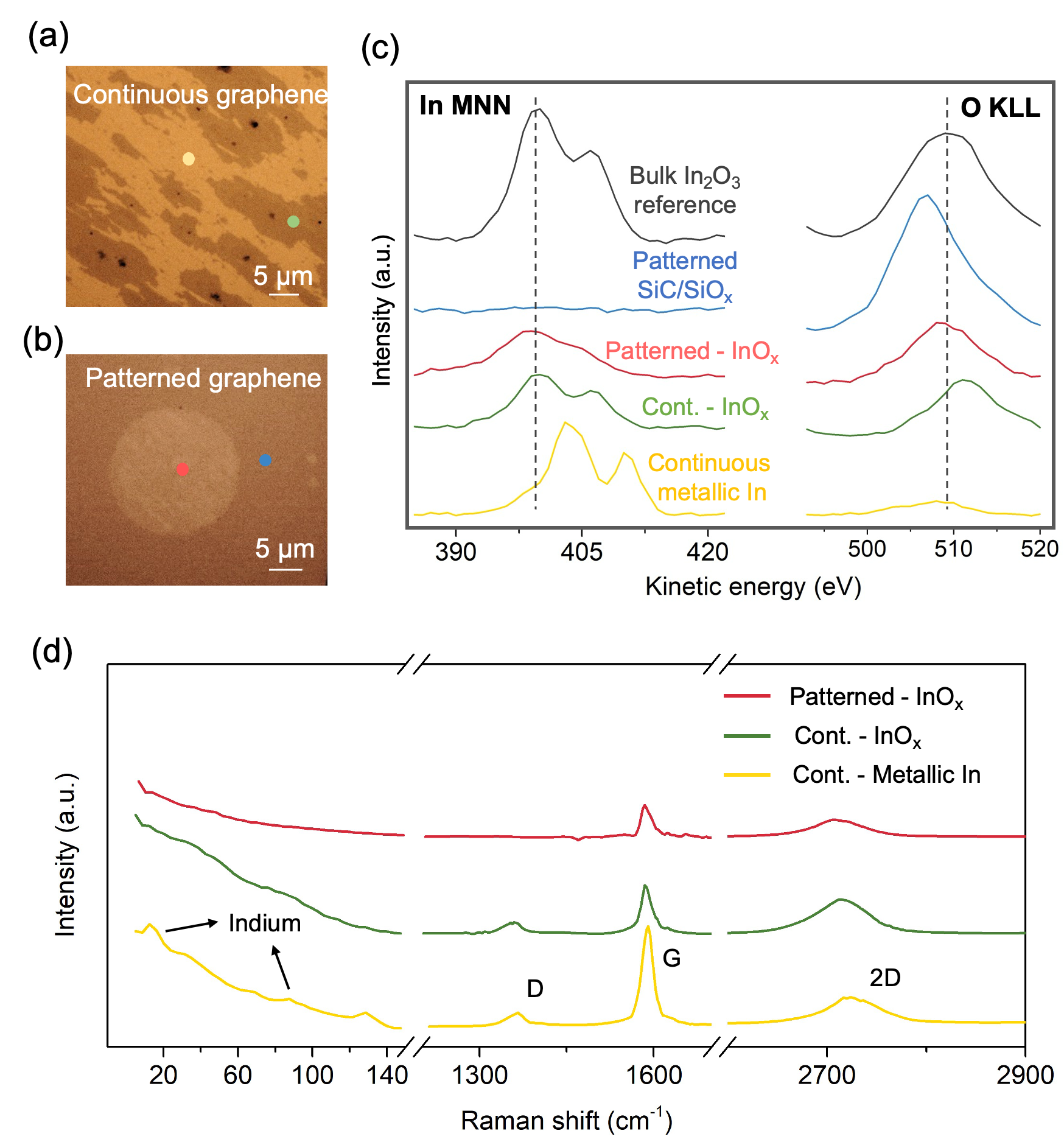


**Figure S6:** Optical micrographs of the indium intercalated continuous (a) and patterned (b) graphene samples after oxidation. Auger spectra (c) from selected regions in these samples, as shown in (a, b). Raman spectroscopy of InO_x_ intercalated continuous and patterned graphene samples (d). Color code in Auger and Raman spectra correspond to the regions shown in (a, b).

Reduction in the indium thickness to monolayer following the oxidation indicate indium deintercalation when patterned graphene is used. This occurs through the outward lateral diffusion of indium to the edges of the patterned graphene circles, as bulk In_2_O_3_ particles are observed at the perimeter of the circles (Figure S7).


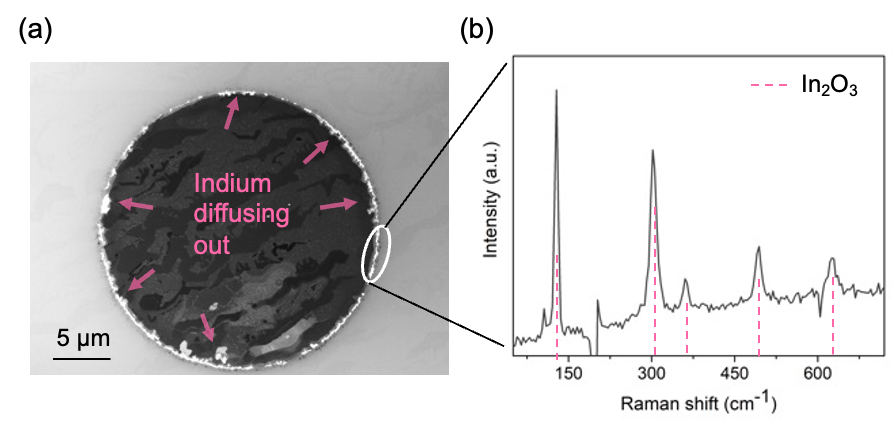


**Figure S7:** Post-oxidation SEM image from EG/InO_x_/SiC with patterned sample (a), demonstrating lateral outward indium diffusion during oxidation. Raman spectrum taken from the particles at the perimeter of the graphene pattern confirms the In_2_O_3_ particles formation due to indium segregation (b).

Surface root mean square (RMS) roughness (R_q_) of the oxidized samples grown using continuous and patterned graphene are distinctly different with 1.99 ± 0.25 nm and 0.52 ± 0.02 nm, respectively. AFM image acquired on the continuous sample shows agglomerated InO_x_ particles on the graphene surface, which is a result of indium deintercalation during oxidation through graphene defects (Figure S8a). Post-oxidation graphene Raman D to G band intensity ratio (I(D)/(IG)) is 0.36 ± 0.06 and 0.07 ± 0.03 for continuous and patterned samples, respectively (Figure S6d). This is not surprising as continuous graphene was treated with low power O_2_ plasma prior to indium intercalation. Although graphene is healed during intercalation,^[2]^ it does not heal fully as I(D)/(IG) ratio after intercalation is 0.15 ± 0.04. These defects may allow indium deintercalation during oxidation as evidenced from the InO_x_ particle formation on the graphene surface (Figure S8a). On the other hand, in patterned sample, graphene remains high quality pre-oxidation as plasma treatment is not applied for intercalation. Although indium deintercalates through patterned graphene/SiC interface as well, this mostly occurs laterally to the edges of the patterns, as it will be discussed later.


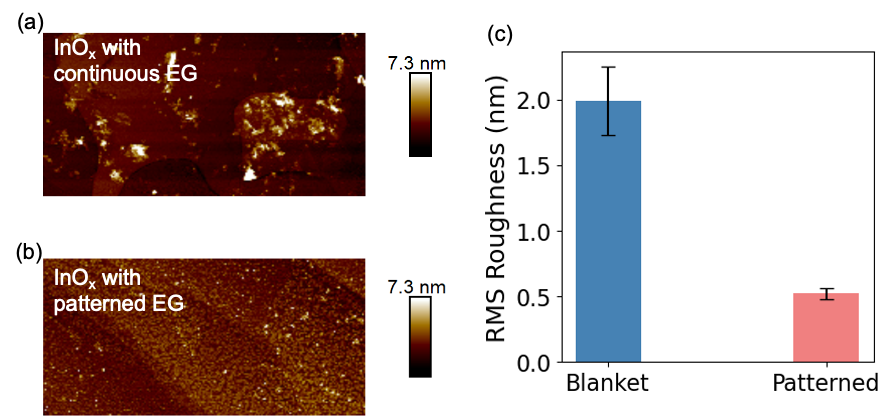


**Figure S8:** Atomic force microscope images of the InO_x_ intercalated continuous (a) and patterned (b) graphene samples. Root mean square roughness of the graphene surfaces is given in (c), verifying smoother surface with patterned sample due to suppressed indium deintercalation through graphene defects, as well as lateral outward diffusion of In to the perimeter of the circular pattern.

# ReaxFF Molecular Dynamics Simulations


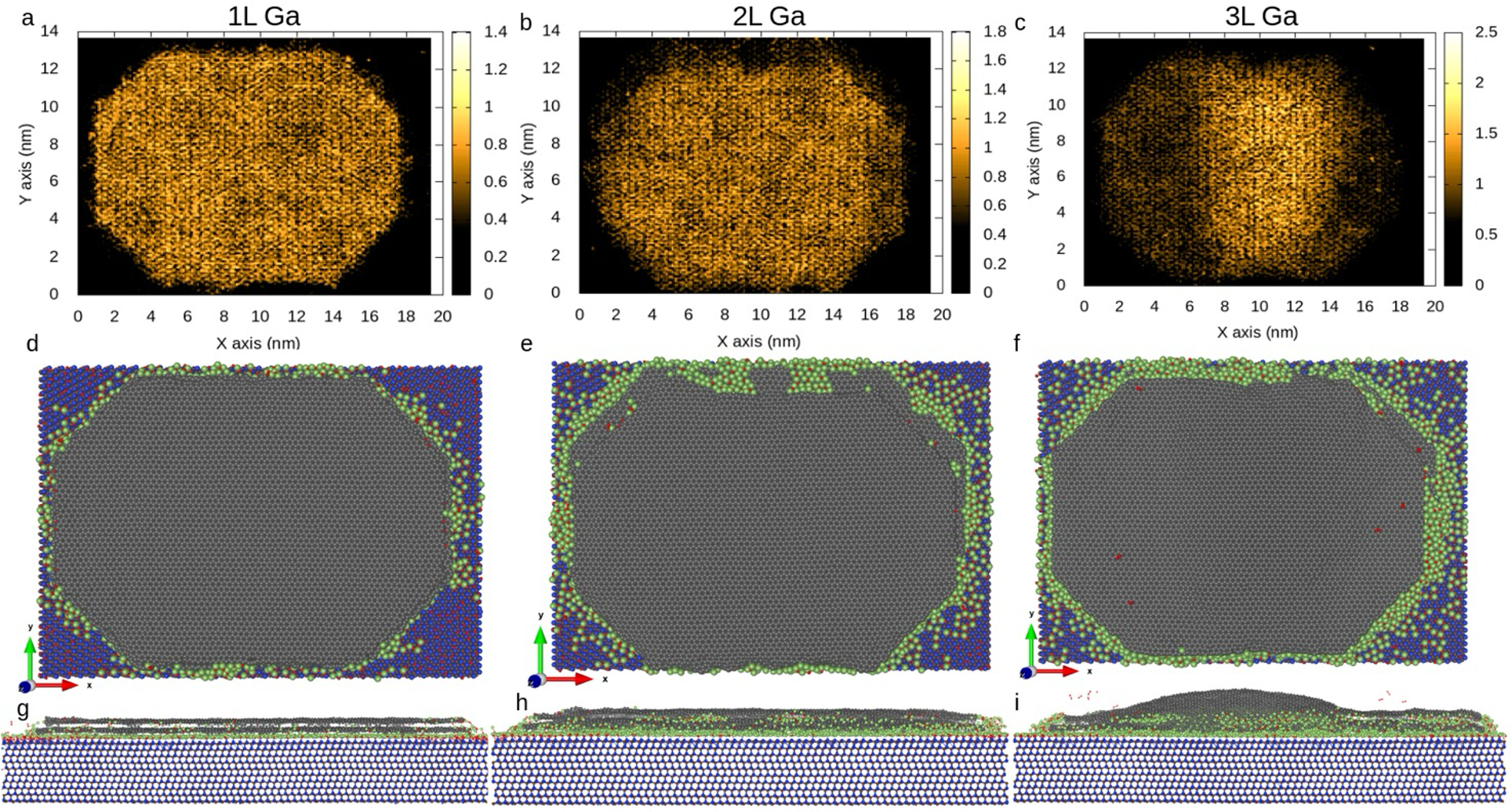


**Figure S9:** Heat maps of the SiC/bilayer graphene models with (a) one Ga (b) two Ga and (c) three Ga layers annealed at 1500K for 150 ps. The brighter regions represent the positions of Ga within the models. Top and side views of the models (d, g) in a, (e, h) in b and (f, i) in b, respectively. Si, C, Ga and O are represented by blue, gray, green and red balls, respectively.


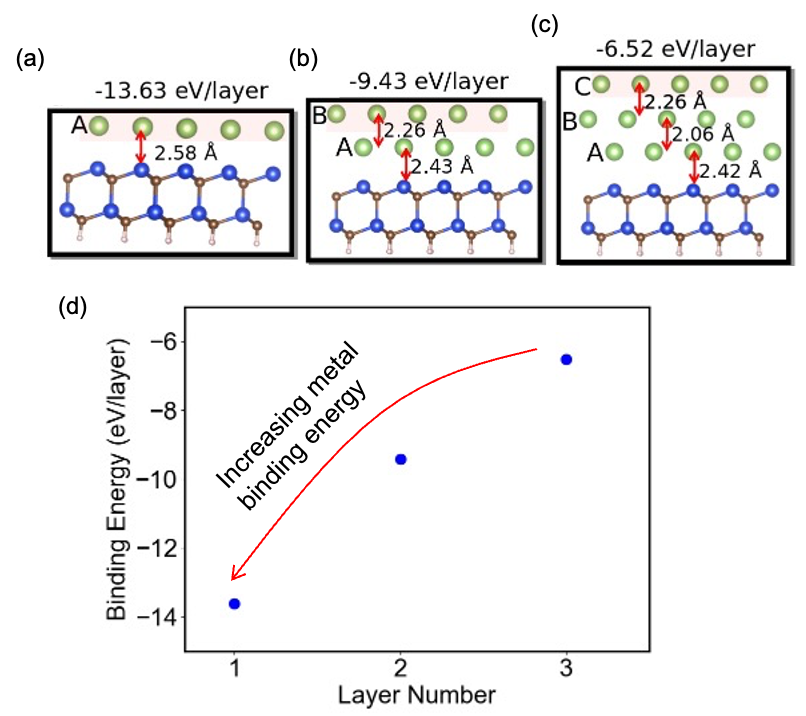


**Figure S10:** Atomic illustrations of the models used in DFT calculations. Side and top views of the models with (a) one, (b) two and (c) three Ga layers deposited on a Si-terminated 6H-SiC(0001) with bottom saturated by H atoms. Ga atoms on the first layer in a deposited on the top site of surface Si atoms, with the second in (b) and third layers in (c) following an ABC stacking sequence starting from the substrate surface. Binding energy vs Ga layer number plot (d). The binding energies of a Ga layer with the SiC surface and other Ga-layers are displayed at the top of each subfigure. Si, C, Ga and O are represented by blue, brown, gray and red balls, respectively.

# Structure of 2D InO_2_

**Table S1.** Structural stability calculations via DFT, with indium and oxygen atomic positions projected onto silicon (Si), hollow (H), and carbon (C) sites of SiC. Calculations yield nearly energetically degenerate two structures with Si-H-C and Si-C-H stacking sequence. In atomic structures, Si is represented by blue, C by brown, O by yellow, and In by red colors.

| **Bottom Oxygen**  **Position** | **Indium**  **Position** | **Bottom Oxygen**  **Position** | **Relative Stability**  **(eV/indium)** | **SiC [11-20]**  **view** |
| --- | --- | --- | --- | --- |
| Silicon | Hollow | Carbon | 0 (Ref) | 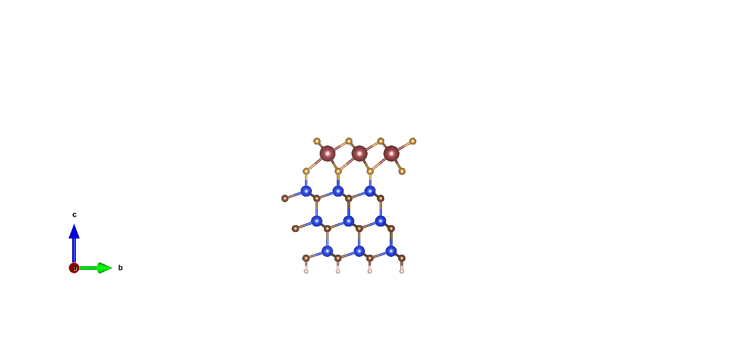 |
| Silicon | Hollow | Silicon | +0.36 | 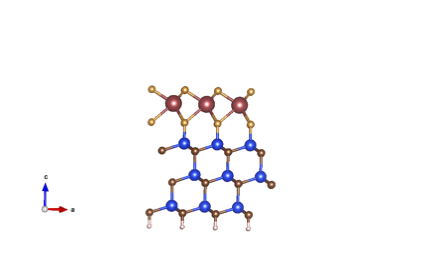 |
| Silicon | Carbon | Silicon | +0.37 | 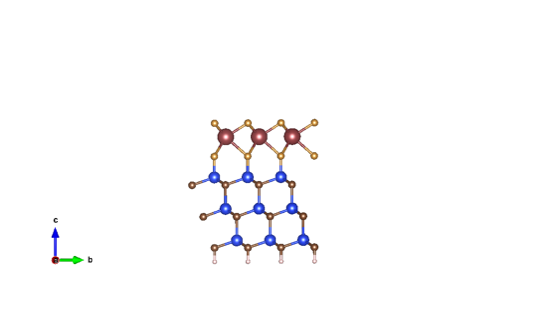 |
| Silicon | Carbon | Hollow | -0.01 | 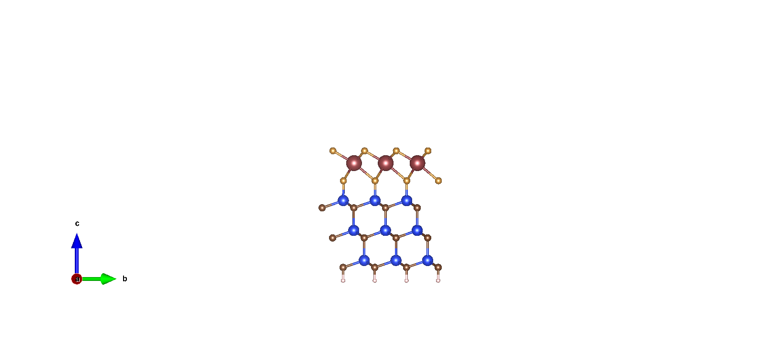 |

To verify Gr-O bonding, we created 44 crystal structures using a 6x6 supercell of the SiC slab, intercalant, and graphene, by modifying the lowest energy monolayer InO_2_ structure with Si-H-C stacking sequence. These structures include InO_2_ with varying concentrations of indium vacancies (2/3, 3/4, 4/36, or 3/36 missing indium atoms), oxygen vacancies, excess oxygen, and no vacancies at all. The formation of Gr-O bonds is anticipated by positioning the graphene sheet close to the top oxygen layer. Additional structures were also simulated for specific purposes, to be explained later. All structures were then relaxed through ab initio DFT as described in the methods section. We found that Gr-O bonding becomes energetically favorable in the presence of an indium vacancy (1.57 eV/defect). Specifically, two to three Gr-O bonds are likely to form where there is one indium vacancy. This can be rationalized by considering the formal charge of the system: when graphene bonds with oxygen, the formal charge of the oxygen increases from 2- to 1-, while the carbon's formal charge remains unchanged in low energy structures by altering its hybridization and reducing the number of double bonds, thereby avoiding the creation of high-energy carbon radicals. As the formal charge of the system increases due to Gr-O bonding, compensation is achieved by either adding excess oxygen or creating indium vacancies. Structures without vacancies start with zero formal change, hence simulation data indicates that the formation of Gr-O bonds is energetically unfavorable, as it would increase the formal charge to positive. The same is true for structures with missing oxygen, which already begin with a positive formal charge prior to forming Gr-O bonds (+2.16 eV/defect compared to those with an indium vacancy). The results for the relaxed structures with Gr-O bonding and either excess oxygen or an indium vacancy are detailed below.

**Excess oxygen:** A structure with excess oxygen starts out with negative formal charge. Such structure with Gr-O bonds is indeed lower in energy than the same structure without Gr-O bonds. However, simulation suggests that excess oxygen would break commensuration and result to disordered structure that is not observed in most part of the sample. This is likely due to the chemical potential cost of having excess oxygen atoms under graphene instead of free oxygen gas.

**Indium vacancy:** A structure with missing indium starts out with 3- formal charge, so it is favorable to increase its formal charge to be closer to zero by forming two to three Gr-O bonds, i.e. Gr-O bonding becomes energetically favorable in the presence of an indium vacancy by 1.57 eV/defect. An indium vacancy creates a -3 formal charge as it has three oxygen atoms as nearest neighbors. In geometric relaxations, two or three Gr-O would spontaneously form to change the formal charge to -1 or 0. The reason why only two Gr-O bonds are formed in some cases, is because of the geometric misalignment between the third oxygen and the closest carbon. In some cases, another reason of not forming the 3rd bond is to avoid creating energetically unfavorable radical/unpaired electron. Additionally, a clustering of indium vacancies and Gr-O bonds is energetically more favorable than a sparse and uniform distribution of indium vacancies by 0.47 eV/defect (Figure S11). This likely stems from the fact that regions with and without Gr-O bonds prefer shorter and larger interlayer distances between the oxygen and graphene layers, respectively. Clustering Gr-O bonds reduces the energetic cost associated with these compromising interlayer distances. Additionally, STEM intensity profiles along the z-axis for the simulated structure with clustered In vacancies and Gr-O bonds (Figure S11c, d), and the experimental STEM images, match each other, as shown in Figure 2h in the main text, demonstrating that the simulated structure in Figure S11c, d could be the experimental product.

Instead of using two or three Gr-O bonds to compensate for the 3- formal charge from missing indium, we also considered, for instance, using one Gr-O bond (1+ formal charge) and one missing oxygen (2+ formal charge). We created more structures with various numbers of Gr-O bonds and missing/escaped oxygen which are placed above graphene sheet as free oxygen gas. However, forming only Gr-O bonds without missing/escaped oxygen is still more favorable by ~2.16 eV/defect. Further entropic contribution of free oxygen gas in typical experimental condition is way smaller than this energy scale. Therefore, we expect the Gr-O bond formation is due to In vacancies.


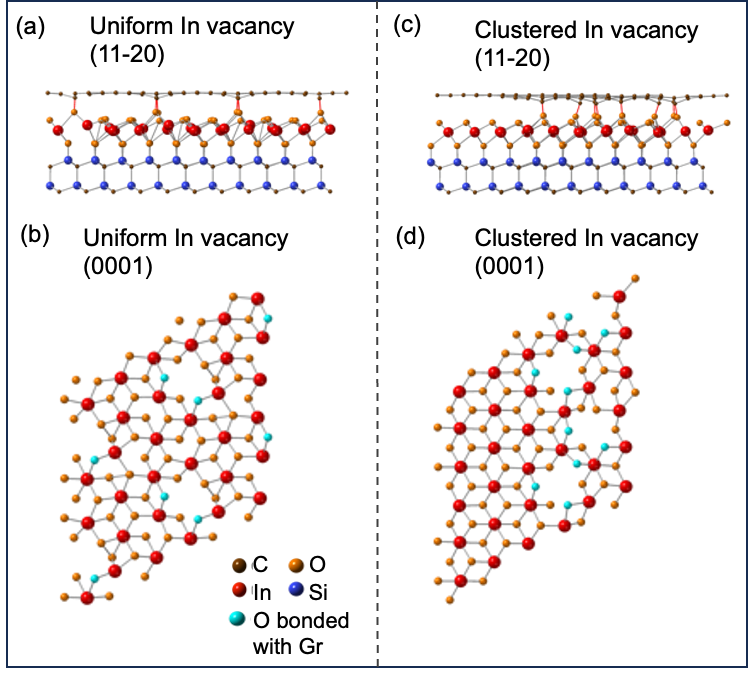


**Figure S11:** Lowest energy monolayer InO_2_ structures with 4/36 indium vacancies and Gr-O bonding. (11-20) and (0001) views of structures with uniform (a, b) and clustered (c, d) In vacancy and Gr-O bonding distribution. Clustering of indium vacancies is 0.47 eV/defect more energetically favorable than uniformly distributed indium vacancies. O atoms bonded with C in graphene are highlighted in turquoise.

# Electronic and Phonon Band Structure Modifications in EG/InO_2_/SiC


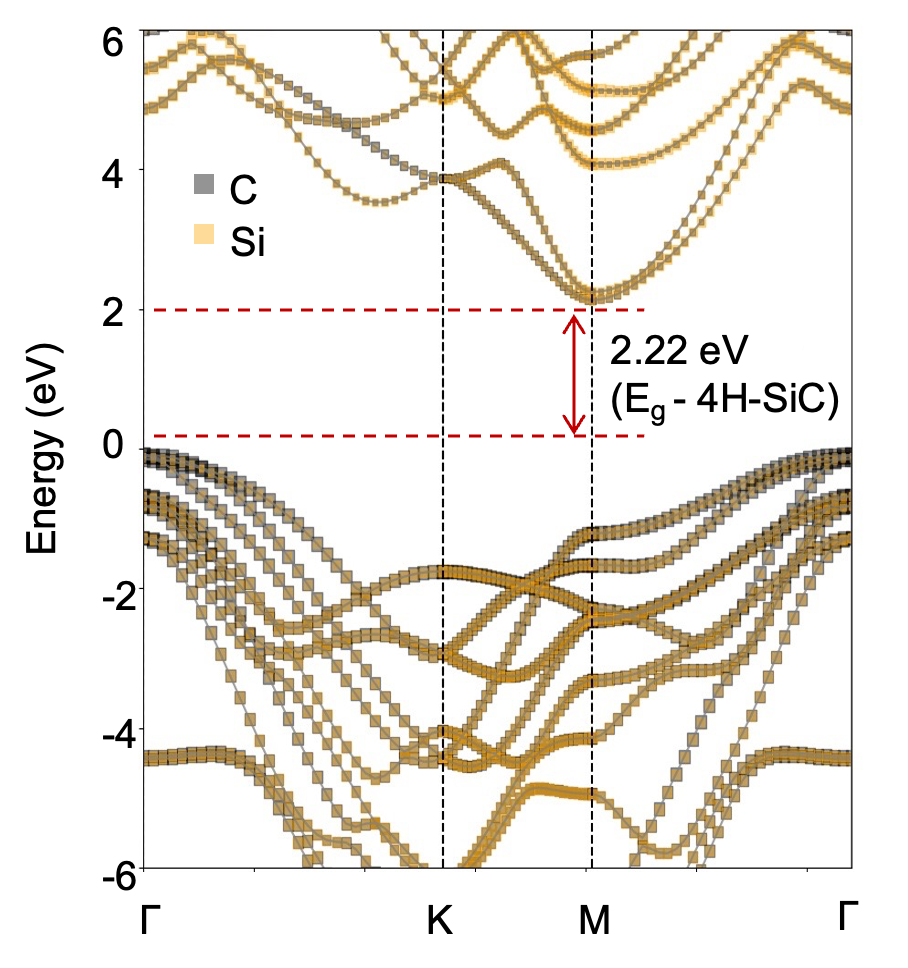


**Figure S12:** Calculated band structure of pristine 4H-SiC, demonstrating underestimated bandgap of 2.2 eV (1 eV lower than the experimental value).


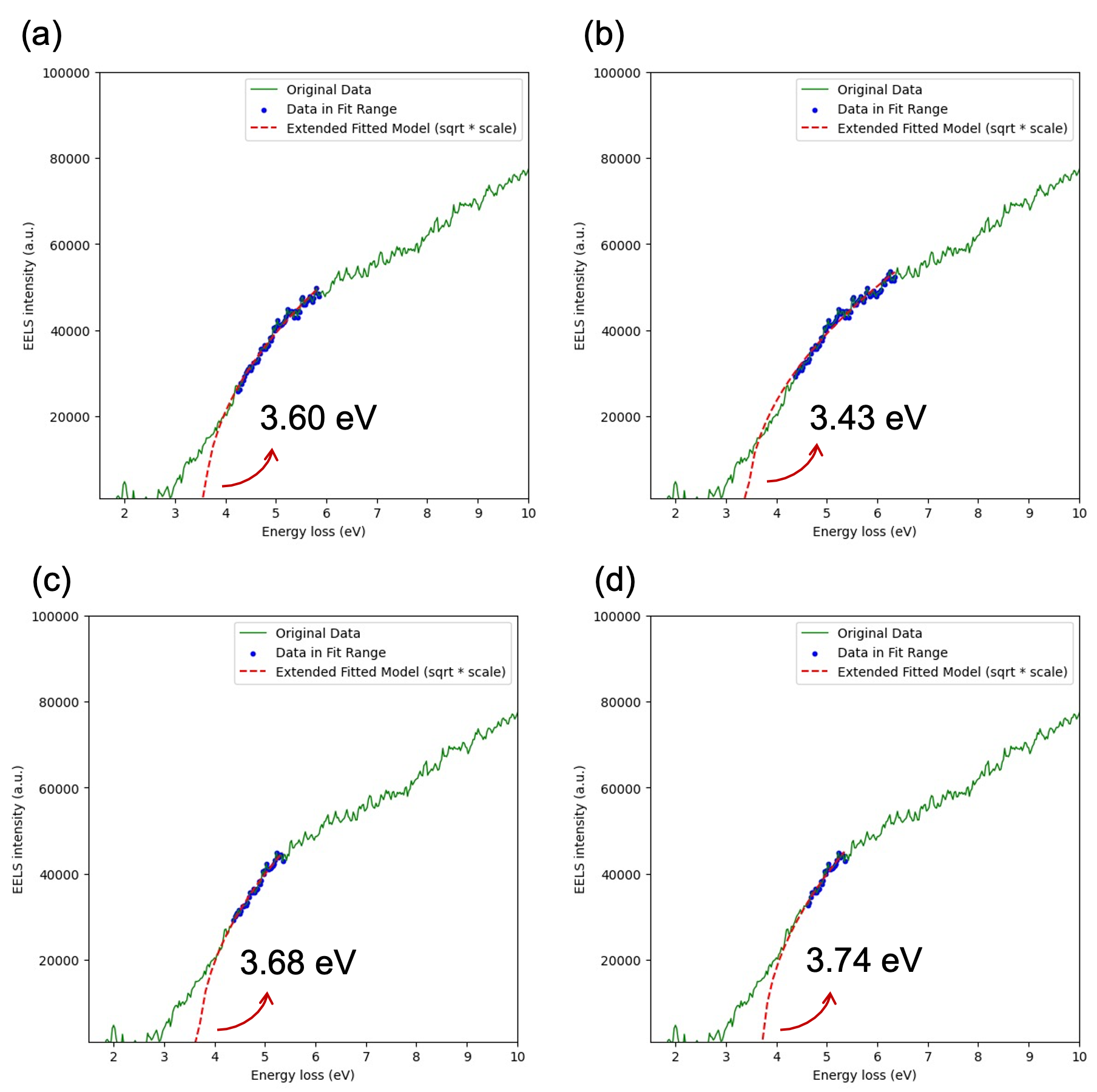


**Figure S13:** Low-loss EELS spectrum of EG/InO_2_/SiC heterostructure with power law fitting (E-E_g_)^0.5^. Extracted absorption energy highly depends on the chosen energy window for the fit, as variations in this window yielded fit results ranging from 3.43 to 3.74 eV.

The structural modifications of InO_2_ in 2D also impact its phonon band structure. Figure S14a demonstrates the Raman spectra taken from In, InO_2_ and mixed In/InO_2_ intercalated EG/SiC. Upon full oxidation, indium ULF peaks (17, 45, and 96 cm^-1^) disappear (Figure S14a). On the other hand, in partially oxidized sample, in addition to the residual ULF peaks, 2D InO_2_ peaks emerge at 295, 323, 421, and 450 cm^-1^, likely due to surface enhanced Raman scattering (SERS) effect when near metallic indium. Auger spectroscopy confirms this partial oxidation; the peak for metallic indium shifts from 403 eV to 399.8 eV when fully oxidized, similar to bulk In_2_O_3_ (Figure S14b). Partially oxidized samples show a peak at 401.8 eV with a shoulder at 403 eV, indicating some metallic indium remains.


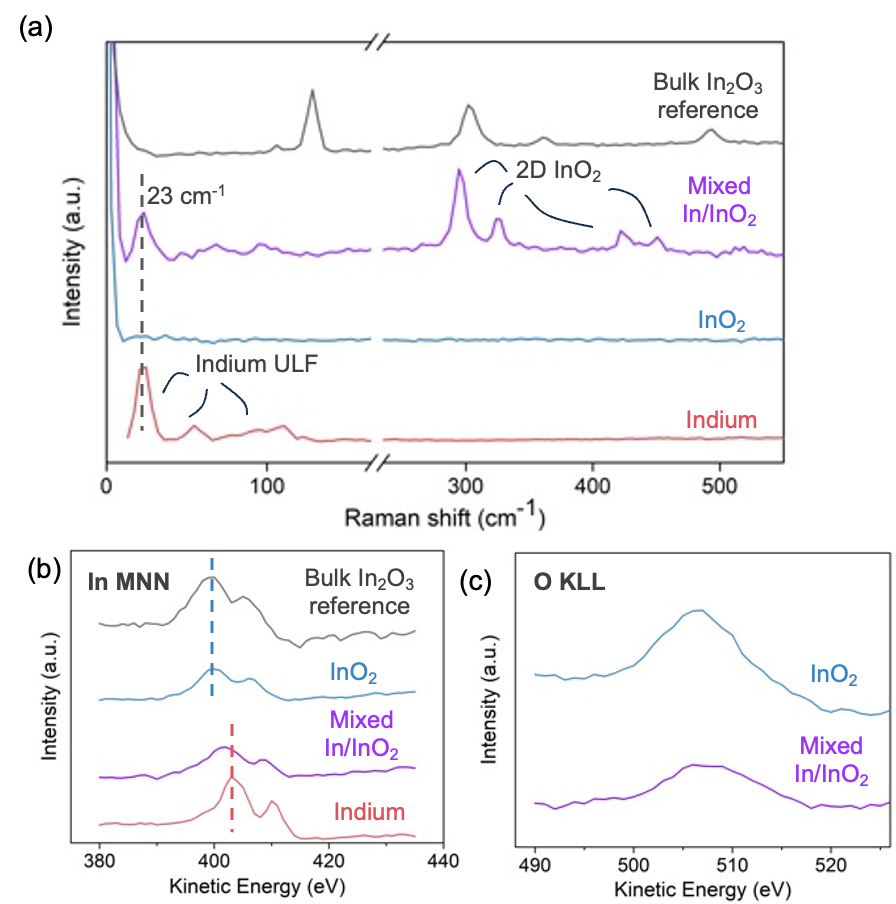


**Figure S14.** Raman (a) and Auger In MNN (b), O KLL (c) spectra taken from indium, InO_2_, and mixed In/InO_2_ intercalated EG/SiC and bulk In_2_O_3_ particles. “Mixed In/InO_2_” sample refers to partially oxidized EG/In/SiC at 600 ^o^C for 15 min and demonstrates 2D InO_2_ Raman bands.

To investigate the origin of the Raman peaks observed after oxidation the phonon band structure of the EG/InO_2_/SiC heterostructure was calculated where the structure in Figure S11c, d was used. Before discussing our findings, we acknowledge the inherent challenges in achieving precise agreement between experimental and calculated Raman intensities, especially for resonant Raman spectra, as discussed in the literature.^[4,5]^ Discrepancies often arise due to factors such as excitonic effects, anharmonicity, and variations in experimental conditions. Given these limitations, we interpret the calculated intensities primarily as qualitative indicators of Raman activity, confirming the presence of active modes under the specific backscattering and laser polarization conditions rather than aiming for an exact replication of experimental intensities. Figure S15 shows the Raman spectra of the 2D InO_2_ system, comparing experimental and theoretical results. The black line represents the experimental Raman spectrum of an encapsulated 2D InO_2_ system, with peak positions determined by fitting Lorentzian to the raw data summarized in Table S2. The green line shows the calculated Raman spectrum of an idealized structure designed to approximate the experimental sample. The green vertical bars highlight calculated Raman peaks that lie within 15 cm^−1^ of the experimental peak positions, see Table S2. The red dashed line depicts the calculated Raman spectrum of bulk In_2_O_3_. While low-frequency peaks are observed experimentally in metallic In and mixed In/InO₂ systems, several such peaks are also present exclusively in the 2D InO₂ system but absent in bulk In₂O₃. Thus, these low-frequency modes can serve as fingerprints of the 2D nature of the system.


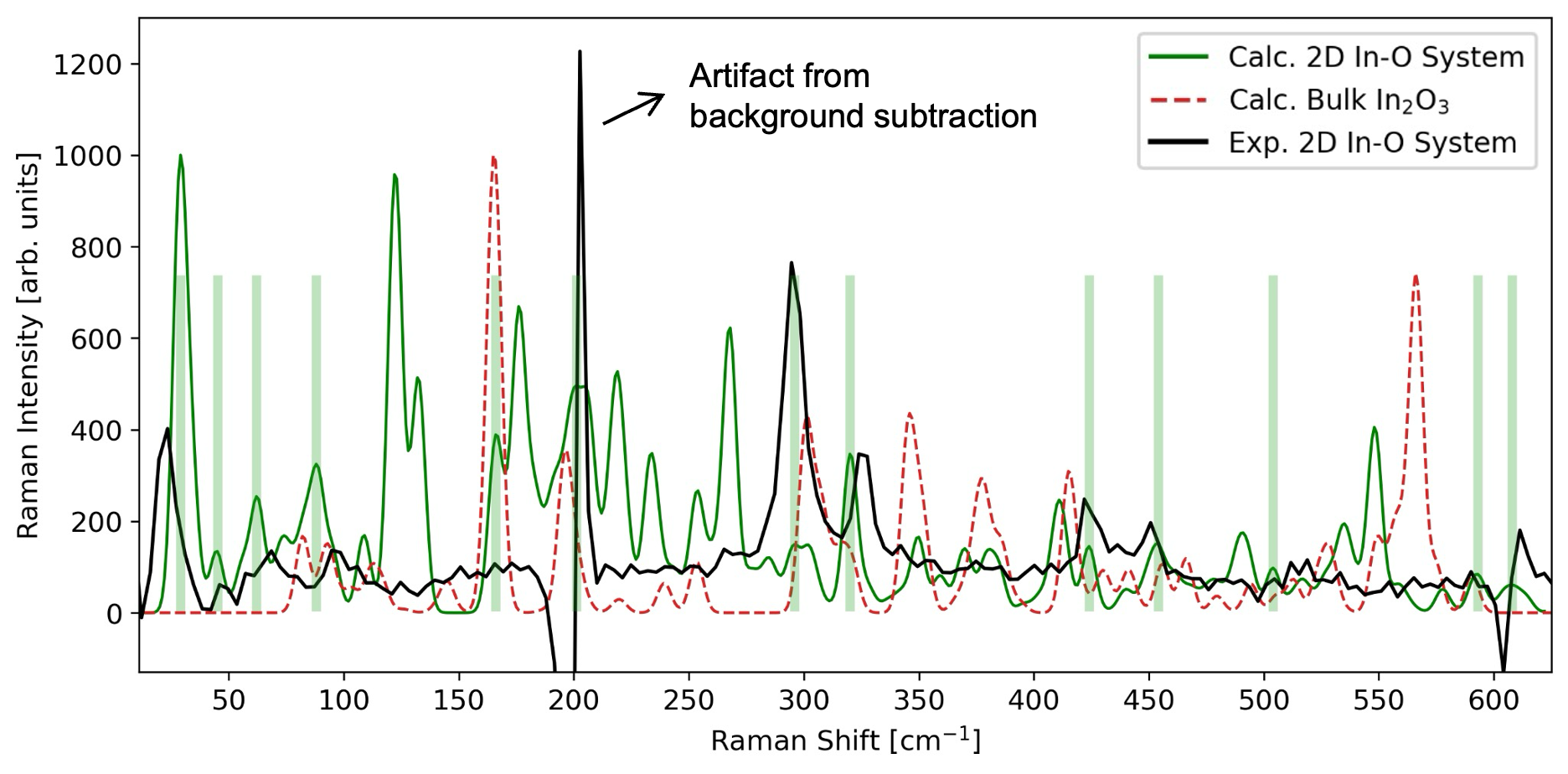


**Figure S15:** Experimental and calculated Raman spectra of the 2D InO_2_ system, with the experimentally observed spectrum shown as a black line and the calculated spectrum as a solid green line. The red dashed line represents the calculated Raman spectrum of bulk In_2_O_3_. Green vertical bars indicate the positions of calculated Raman peaks that fall within 15 cm^-1^ of the corresponding experimental peaks. The exact peak positions are summarized in Table S2.

**Table S2:** Peak positions extracted from fits of the experimental spectrum, compared to calculated peak positions within 15 cm⁻¹ of the experimental values. Lorentzian fits to the experimental Raman peaks were used to determine the experimental peak positions. To initialize the fitting process, the raw data was analyzed using the find_peaks function from scipy.signal, with a prominence threshold set at 3% relative to the baseline background.

| **Peak label** | **Experiment [cm^-1^]** | **Calculation [cm^-1^]** |
| --- | --- | --- |
| P1 | 22.8 | 29 |
| P2 | 51 | 45 |
| P3 | 68.6 | 62 |
| P4 | 97.1 | 88 |
| P5 | 168 | 166 |
| P6 | 203 | 201 |
| P7 | 295.8 | 296 |
| P8 | 324.7 | 320 |
| P9 | 424.7 | 424 |
| P10 | 448.7 | 454 |
| P11 | 517 | 504 |
| P12 | 587.8 | 593 |
| P13 | 614.4 | 608 |

We can understand the unique 2D nature of the low-frequency peaks by visualizing the underlying atomic motions responsible for the peaks. Figure S16a illustrates the atomic dis- placements associated with the P1 Raman mode, calculated at 29 cm^−1^. The structure represents an encapsulated 2D InO_2_ system, with atoms color-coded as follows: brown (C atoms), blue (Si atoms), red (O atoms), and light purple (In atoms). The green arrows indicate the vibrational eigenvectors, showing the directions of atomic motion. This low-frequency mode primarily involves out-of-plane oscillations of the oxygen-coordinated In atoms, coupled with distortions in the underlying 2D framework. The encapsulating graphene layer exhibits minimal displacement, suggesting that the motion is localized within the InO_2_ system. The dominance of out-of-plane motion in this mode aligns with the low-frequency Raman peaks serving as fingerprints of the 2D nature of the system as the interfacial force constants between the In-O system and the encapsulated graphene layer are being probed during this vibration. Similarly, the vibrational modes responsible for peaks P2-P4 (low-frequency modes) primarily involve atomic displacements within the InO_2_ layer with significant out-of-plane displacement components of the In-atoms eigenvectors. Higher-frequency modes behind P5 and the following bands on the other hand, increasingly involve in-plane motions of In and other atoms including those in the graphene top or the SiC bottom layers.


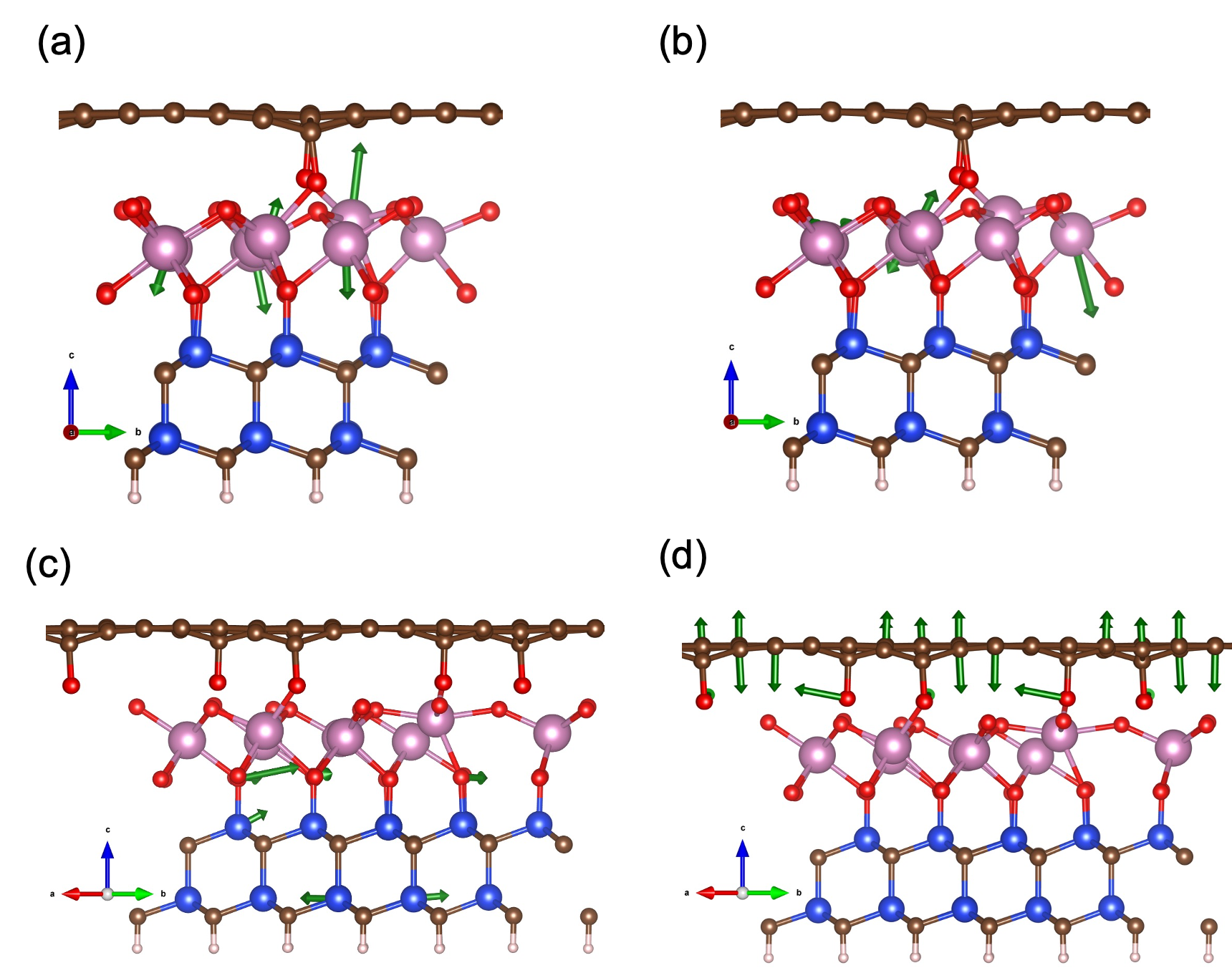


**Figure S16:** Visualization of the highest intensity phonon modes in InO_2_, where panels (a), (b), (c), (d) demonstrate modes denoted as P1, P3, P7, P8 in Table S2.

Compared to the bulk In_2_O_3_ Raman peaks at 303, 360, and 492 cm-1,^[6]^ 2D InO_2_ peaks are red shifted which is attributed to the expansion of In-O bonds influenced by Si-O and C-O bonds present. Structural calculations show that the bond distance between the top oxygen and indium increases from 2.05 Å to 2.2 Å due to Gr-O bonding (Figure S17). Likewise, the presence of Si-O bonds increases the bottom O-In distance to 2.25 Å, larger than the one in bulk In_2_O_3_ (2.14 Å),^[7]^ explaining the observed red shift in the Raman spectra due to bond expansion at the EG/SiC interface.


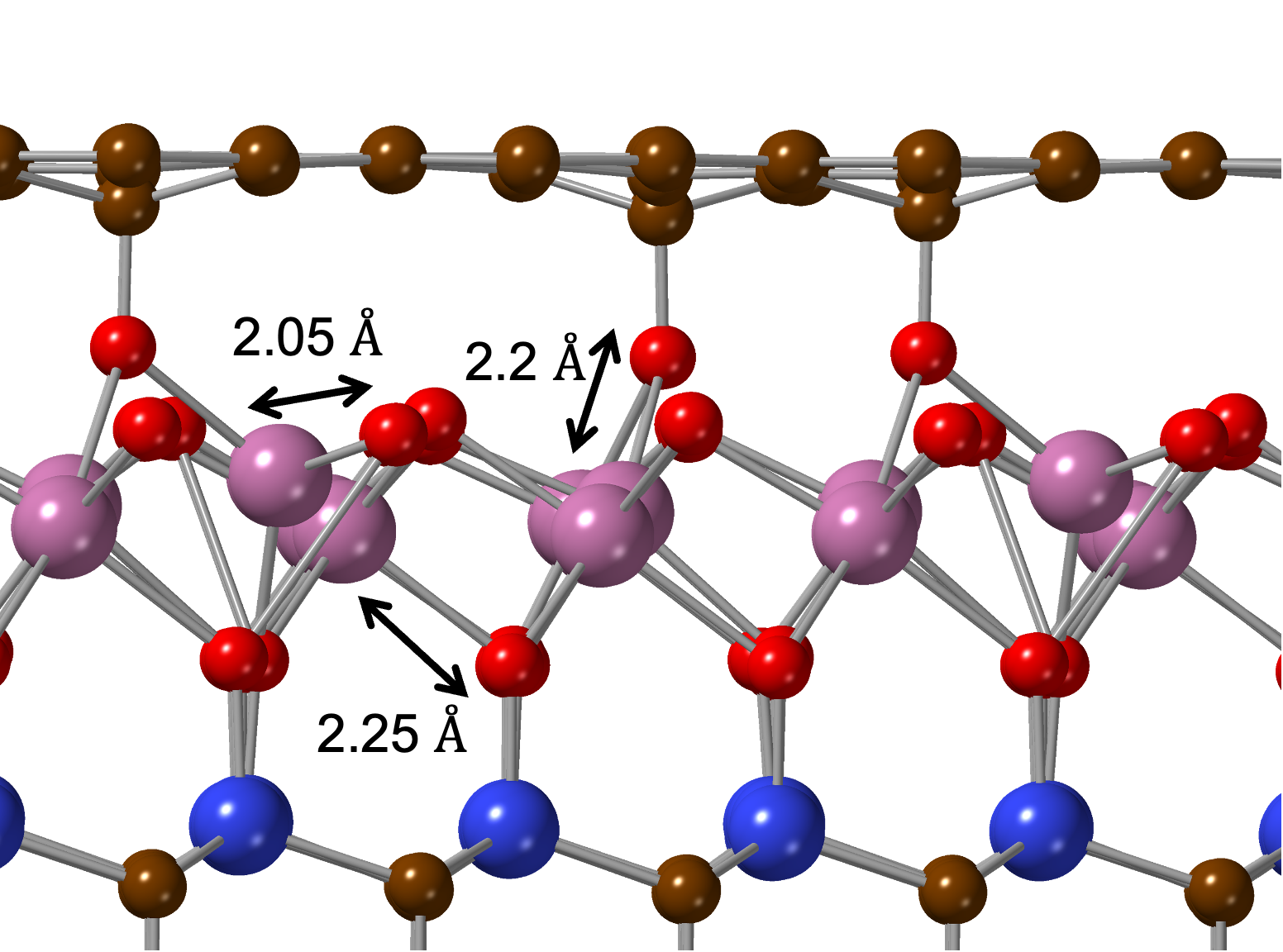


**Figure S17.** Calculated structure of InO_2_ intercalated EG/SiC with Gr-O bonding, demonstrating In-O bond expansion compared to bulk In_2_O_3_ (2.14 Å) due to Gr-O and Si-O bonding. Si, C, In, and O atoms are represented by blue, brown, light purple, and red.

# Indium and InO_2_ Intercalated EG/n-SiC Vertical Diodes

## Device Fabrication

Indium and InO_2_ intercalated EG/n-SiC vertical diodes were fabricated via e-beam lithography (EBL) (Figure S18). First, circular top electrode contact with 0.6 μm diameter was made with Ti/Au (5:40 nm) lift-off on graphene. Then, graphene/InO_2_ heterostructure was etched with O_2_ plasma in 30 seconds to minimize the device size and make sure current does not flow through non-intercalated regions which reduces the threshold voltage and rectification ratio. As an isolation layer, 30 nm Al_2_O_3_ was deposited via atomic layer deposition (ALD) at 150 ^o^C using H_2_O and trimethylaluminum (Al₂(CH₃)₆). Then, contacts were opened by etching Al_2_O_3_ in BCl_3_ and Cl_2_ (30 sccm:10 sccm) plasma. Ti/Au (10:120 nm) leads and pads were defined by lift-off. The bottom contact (n-SiC) was made by depositing Ti/Au (5:40 nm) on multilayer graphene grown on the C face of n-SiC. Finally, the sample was electrically connected to a gold coated AFM stainless steel disks using silver paste to facilitate probe contact. Electrical measurements were conducted with the n-SiC substrate grounded, while voltage was applied to the graphene layer.


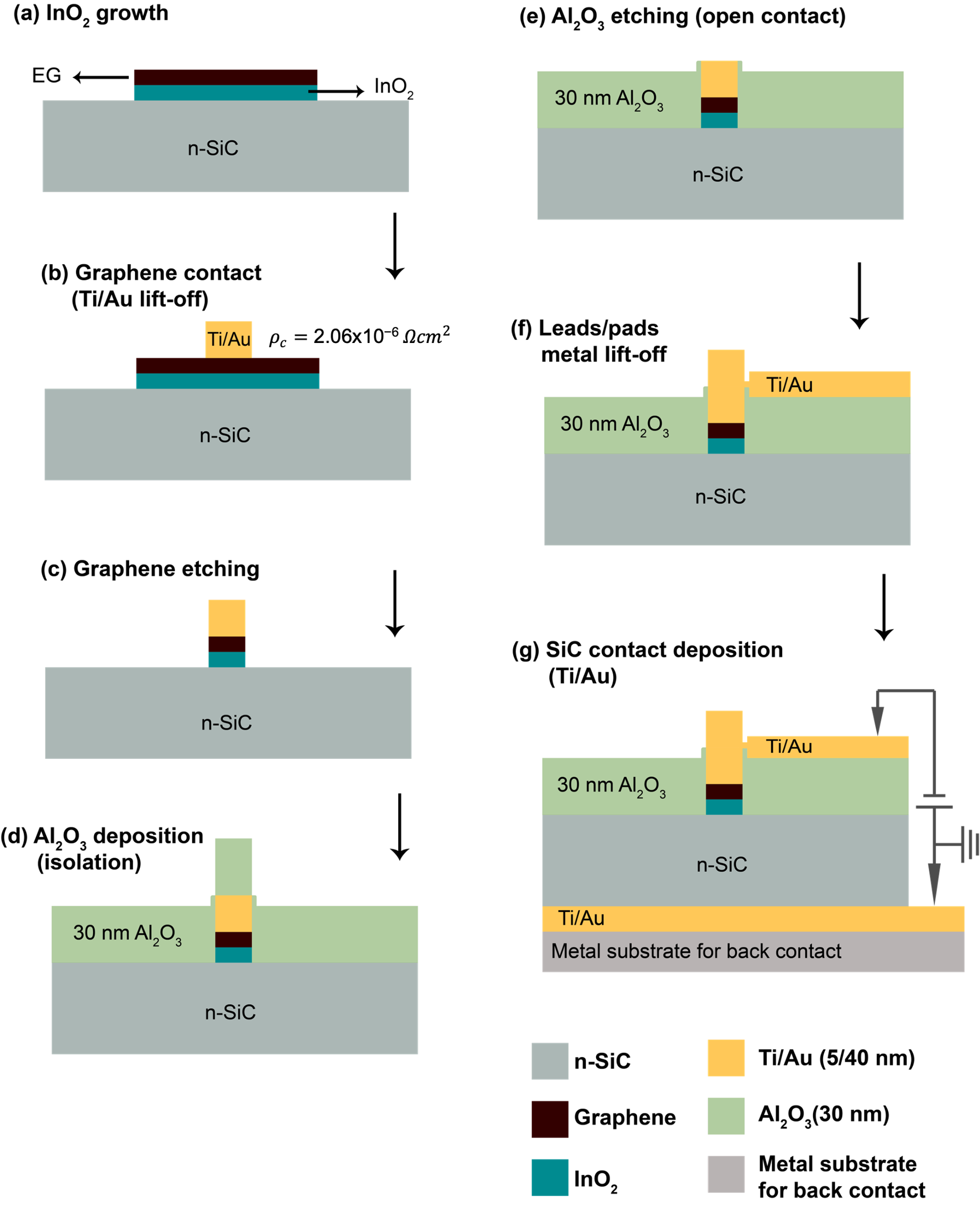


**Figure S18.** Device fabrication steps of the EG/InO_2_/n-SiC diode using e-beam lithography. Top electrode (graphene) contact lift-off with Ti/Au (5:40 nm) (b) following InO_2_ intercalation (a). Top contact has circular geometry with 0.6 μm diameter. Graphene and InO_2_ etching with O_2_ plasma (c). 30 nm Al_2_O_3_ deposition via ALD for isolation between metal leads and n-SiC substrate (d). Al_2_O_3_ etching to open the top contact using BCl_3_/Cl_2_ plasma (e). Ti/Au (10:120 nm) leads and pads lift-off (f). Bottom electrode (n-SiC) deposition with Ti/Au (5:40 nm) at the backside of n-SiC (g). Device has asymmetric geometry with a circular small size top contact (0.6 μm diameter) and 1x1 cm^2^ bottom contact (whole sample).

Device fabrication recipes for both optical and e-beam lithography are given below (Table S3). Prior to spin coating, all the samples were dehydration baked at 150 ^o^C for 3 min and then cooled down to room temperature for 2 min.

**Table S3:** Device fabrication recipes for EG/InO_2_/n-SiC diode via optical and e-beam lithography.

| **Alignment Mark with SiC etching – Optical Lithography** |
| --- |
| Spin LOR5A at 4K, 45 sec. Soft bake at 180 ^o^C, 180 sec. |
| Spin SPR 955 at 3K, 45 sec. Soft bake at 105 ^o^C, 120 min. |
| Expose via optical lithography (Heidelberg: MLA 150) with a dose of 180 mJ/cm^2^, |
| Develop with CD-26 in 75 sec and rinse in DI-water for 60 sec. |
| Etch SiC 550 nm deep via SF_6_ plasma.  Parameters: 20mTorr, 55 sccm SF_6_ (no O_2_), 20 secs etch and 60 sec stabilization for 13 cycles.  Etch rate: 43 nm/cycle. |
| Strip the resist in PRS 3000 at 80 ^o^C in 30 min. Rinse with IPA for 5 min. |
| **Graphene etching (isolation etch) – Optical Lithography** |
| Spin SPR 3012 at 4K, 45 sec. Soft bake at 95 ^o^C, 60 sec. |
| Expose via optical lithography (Heidelberg: MLA 150) with a dose of 200 mJ/cm^2^, |
| Develop with CD-26 in 60 sec and rinse in DI-water for 60 sec. |
| Etch graphene in N_2_ plasma for 15 sec. Antenna power: 200 W, Bias: 10W. N_2_: 20 sccm, |
| Strip the resist in PRS 3000 at 80 ^o^C in 30 min. Rinse with IPA for 5 min. |
| **Top Contact lift-off (Ti/Au:5/40 nm) – E-beam lithography** |
| Spin MMA EL6 (150 nm) at 4K, 45 sec. Soft bake at 150 ^o^C, 90 sec. |
| Spin PMMA 950 A3 (180 nm) at 4K, 45 sec. Soft bake at 180 ^o^C, 90 sec. |
| Expose via EBL (Raith: EBPG 5200 Vistec) - 330 𝜇C/cm^2^ dose. |
| Develop with MIBK/IPA (1:1) for 60 sec and rinse in IPA for 45 sec. |
| Deposit 5/40 nm thick Ti/Au via e-beam evaporation. Dep rate: 0.5 Å/sec for Ti and 2 Å /sec for Au. |
| Lift-off with acetone (50 ^o^C for 15 min) and then PRS 3000 (80 ^o^C for 30 min). Rinse with IPA for 5 min. |
| **Al_2_O_3_ etching – E-beam lithography** |
| Spin Zep (undiluted) at 2.5K, 45 sec. Soft bake at 180 ^o^C, 180 sec. |
| Expose via EBL (Raith: EBPG 5200 Vistec) - 375 𝜇C/cm^2^ dose. |
| Develop with N-amyl acetate for 180 sec and rinse in IPA for 60 sec. |
| Etch Al_2_O_3_ via BCl_3_/Cl_2_ plasma (5 ^o^C, 30 sccm BCl_3_, 10 sccm Cl_2_, 60 secs (4 repetition). Etch rate at 5 ^o^C is 6 Å /sec. |
| Strip the resist in PRS 3000 at 80 ^o^C in 30 min. Rinse with IPA for 5 min. |
| **Metal pads/leads lift-off (Ti/Au:10/120 nm) – E-beam lithography** |
| Spin MMA EL11 at 4K, 45 sec. Soft bake at 150 ^o^C, 90 sec. |
| Spin PMMA 950 A3 at 4K, 45 sec. Soft bake at 180 ^o^C, 90 sec. |
| Expose via EBL (Raith: EBPG 5200 Vistec) - 375 𝜇C/cm^2^ dose. |
| Develop with MIBK/IPA (1:1) for 60 sec and rinse in IPA for 45 sec. |
| Deposit 10/120 nm thick Ti/Au via e-beam evaporation. Dep rate: 0.5 Å/sec for Ti and 2 Å /sec for Au. |
| Lift-off with acetone (50 ^o^C for 15 min) and then PRS 3000 (80 ^o^C for 30 min). Rinse with IPA for 5 min. |
| **Bottom contact (Ti/Au:5/40 nm) – No lithography** |
| Ti/Au (5:40 nm) deposition at the multilayer graphene grown on C face of SiC. |

## Graphene Contact Resistance

We first examined the electrical properties of the graphene/Ti/Au interface to assess the contact resistance of the top electrode. Figure S19a displays room temperature resistance measurements taken from graphene, contacted with Ti/Au (5:40 nm) on insulating 6H-SiC substrate using circular transfer length method (CTLM) with changing electrode spacing d (channel length in Figure S19b). The measurements were taken using four-point probe technique to eliminate resistance originating from the probes. The results confirmed ohmic I-V characteristics across all voltage levels. A geometry correction for CTLM has properly been carried out, following the procedure in Ref ^[8]^. Each point in Figure S19b represents the slope of a linear I-V curve. The linear relationship observed with distance allowed us to calculate the contact resistivity (*ρ_C_* = 2.06x10^-6^ Ωcm^2^), sheet resistance (*R_sh_* = 0.14 Ω/sq) and the transfer length (*L_c_* = 0.39 μm), based on the Equations S1-S3, where *R_T_* is total resistance measured via four-point probe, *R_c_* is graphene/Ti/Au contact resistance, *R_sh_* is sheet resistance, *l* is contact spacing, and *W* is contact area, *L_T_* is transfer length.

**Figure S19:** Electrical properties of graphene/Ti/Au contact using CTLM. Current-voltage curves taken from Ti/Au contacted graphene with various contact spacing, verifying linear relationship (a). Geometry corrected R_T_ as a function of contact spacing l, demonstrating linear relationship (b). Contact resistivity of 2.06x10^-6^ Ωcm^2^ is extracted from the intercept of (b).


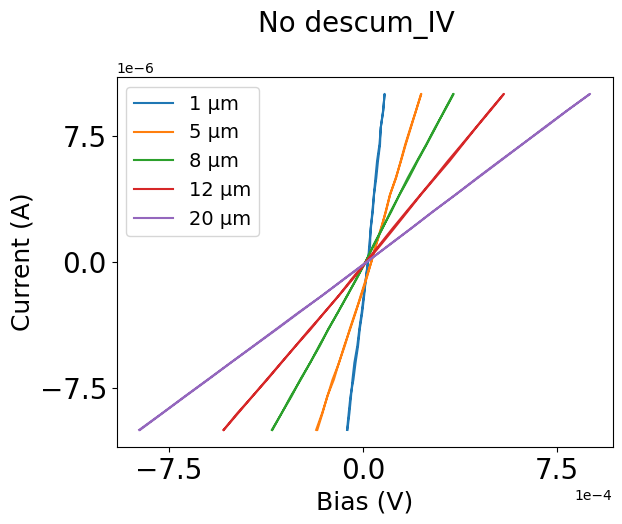

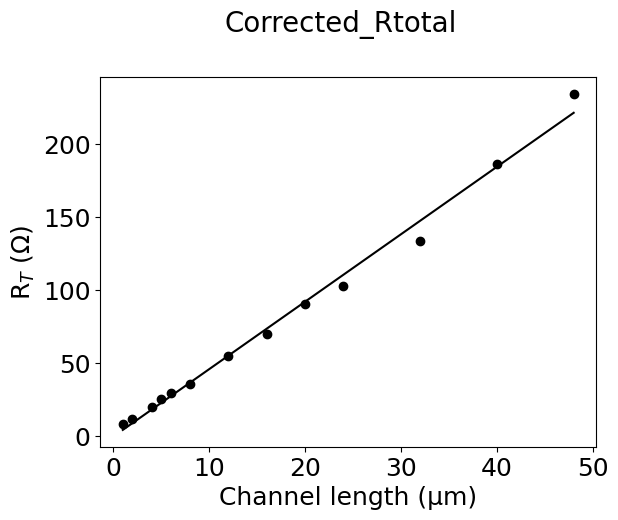

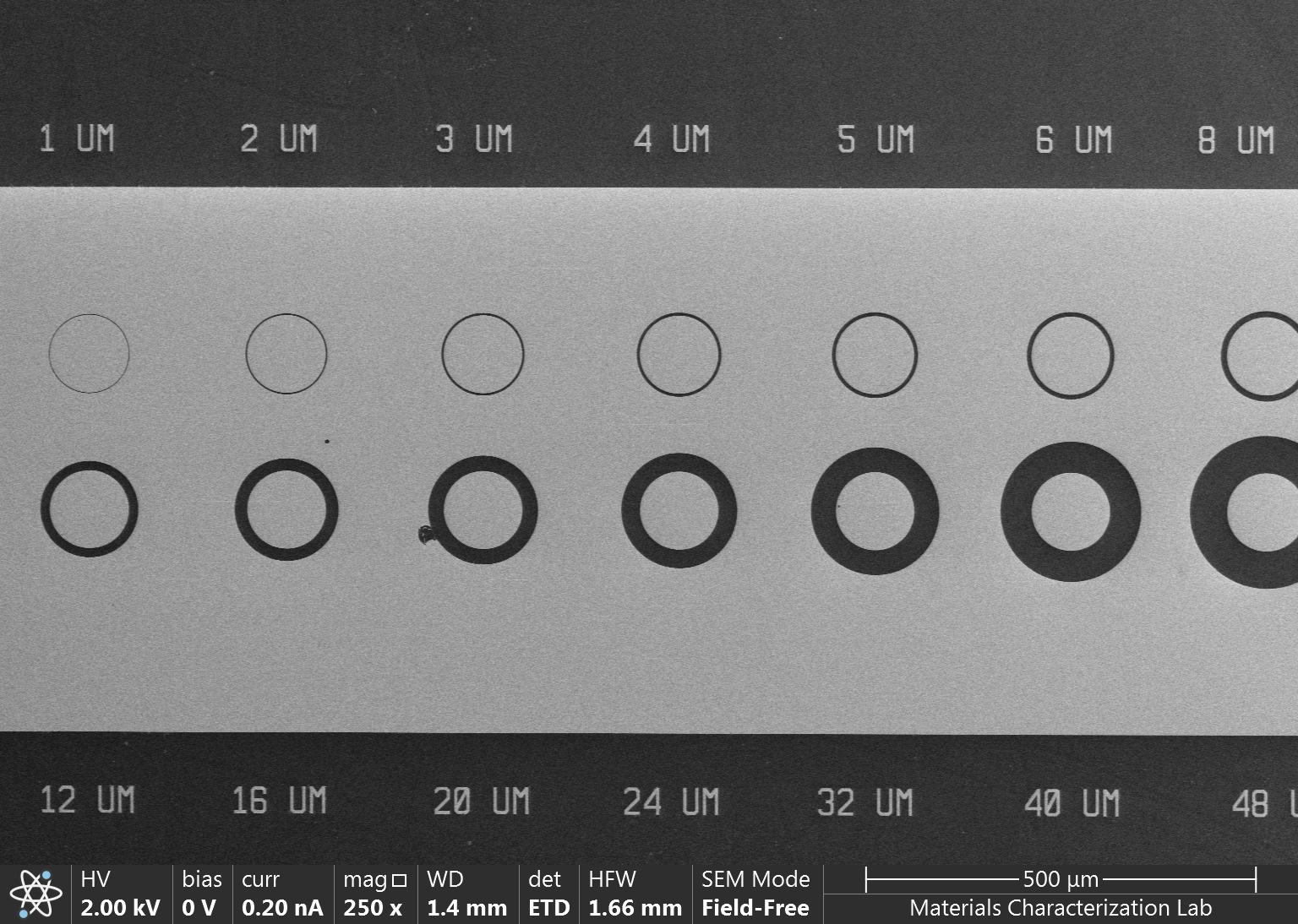


R_sh_ = 0.14 Ω/sq

L_T_ = 0.39 μm

$\rho_{c}$ *(Ωcm^2^) = 2.06x10^-6^*

(a)

(b)

250 μm

Ti/Au

EG

$R_{T}=2R_{c}+R_{sh}\times\frac{l}{W}$ Equation S1

$R_{T}=\frac{R_{sh}}{W}\left( {l+2L}_{T} \right)$ Equation S2

$\rho_{c}=R_{sh}\times{L_{T}}^{2}$ Equation S3

For the bottom electrode we contacted n-SiC from the C face (000-1) by depositing Ti/Au (5/40 nm) via e-beam evaporation without any lithography step (Figure S20a). A common approach to form low resistance ohmic contact to n-SiC is to deposit Ni and anneal at temperatures >1000 ^o^C.^[9]^ As annealing the sample at such high temperatures would lead to deintercalation of 2D oxide, we used the multilayer graphene grown on the C face of SiC to form ohmic contact to n-SiC with Ti/Au. When EG is grown via Si sublimation from SiC, graphene grows on all surfaces, including the backside of the SiC wafer (000-1). This interfacial graphene layer helps for the formation of ohmic contact between n-SiC and metal contact.^[10]^ Figure S20b demonstrates I-V curve taken from Ti/Au contacted as-grown EG/n-SiC (contacted both sides), verifying ohmic conduction for vertical transport (~125 Ω). Hence, observed barrier in diode measurements with intercalated structures is attributed to the modulations at the interface.


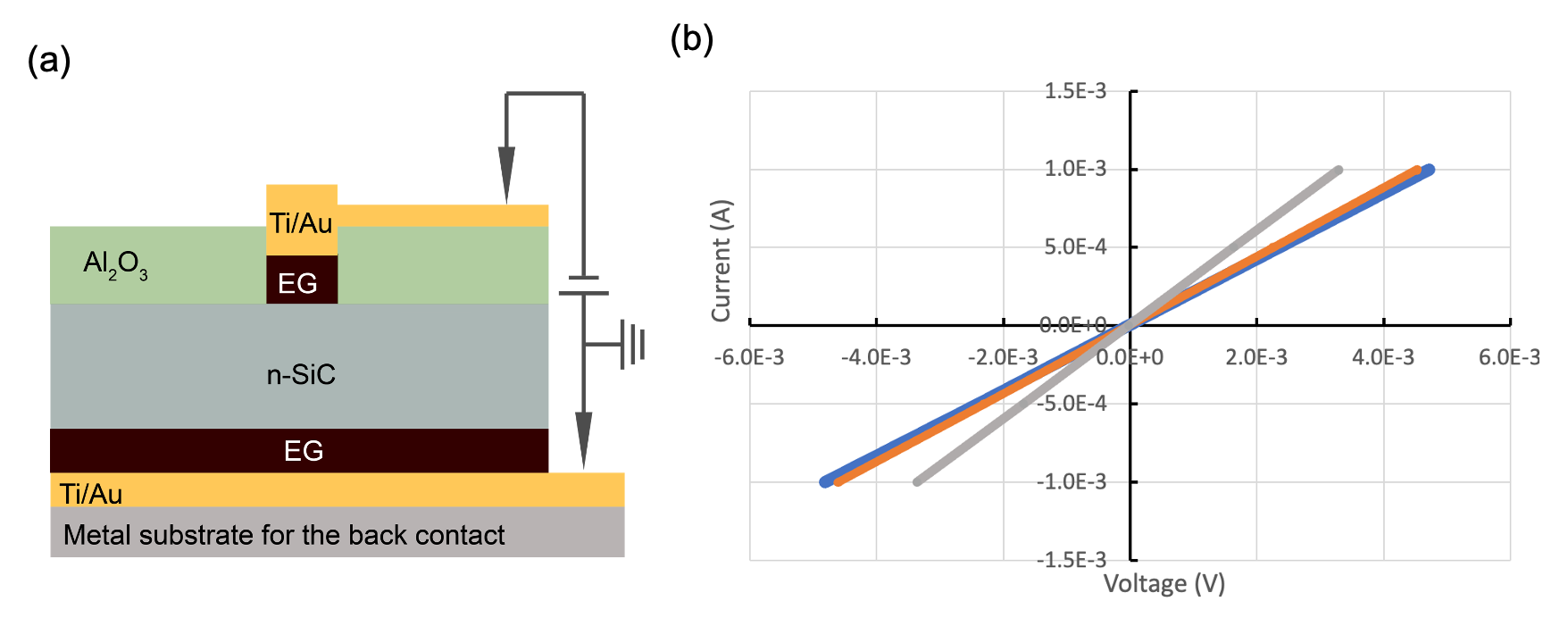


**Figure S20**. Two-terminal vertical device measurements with as-grown graphene/n-SiC contacted with Ti/Au. (a) showing the schematic for the measurement system. (b) I-V curves taken from Ti/Au contacted as-grown EG/n-SiC two terminal devices showing ohmic conduction.

## EG/InO_2_/n-SiC Vertical Diode Measurements

Epitaxial graphene layer that grows on top of buffer layer (first layer of EG) typically exhibits strong n-type doping, which arises from charge transfer from the SiC substrate through the interfacial Si dangling bonds. In this state, the graphene and the SiC substrate are strongly coupled (Figure S21), and the contact often exhibits ohmic-like behavior due to the minimal barrier for electron injection across the interface (Figure S22). Upon intercalation (hydrogen, metal, or compound), intercalant diffuses to the interface between the buffer layer and the SiC substrate. The intercalant atoms break the existing Si–C covalent bonds and saturate the Si dangling bonds, effectively passivating the SiC surface. This process converts the buffer layer into a quasi-free-standing graphene (QFEG) layer, thereby decoupling it electronically from the substrate. The removal of direct chemical bonding drastically reduces charge transfer from SiC to graphene, resulting in a substantial reduction in n-type doping (e.g. QFEG is p-type doped when H is intercalated). Consequently, the Fermi level of graphene moves away from the conduction band edge of SiC, leading to band bending and the formation of a Schottky barrier at the interface (Figure S21). This transformation changes the carrier transport behavior from ohmic-like (dominated by direct tunneling or thermionic field emission) to rectifying (governed by thermionic emission across the Schottky barrier). Hence, intercalation modifies the electronic structure at the graphene/SiC interface, transforming it from a chemically bonded, highly conductive junction into a passivated, semiconducting heterointerface characterized by Schottky barrier formation.^[10]^


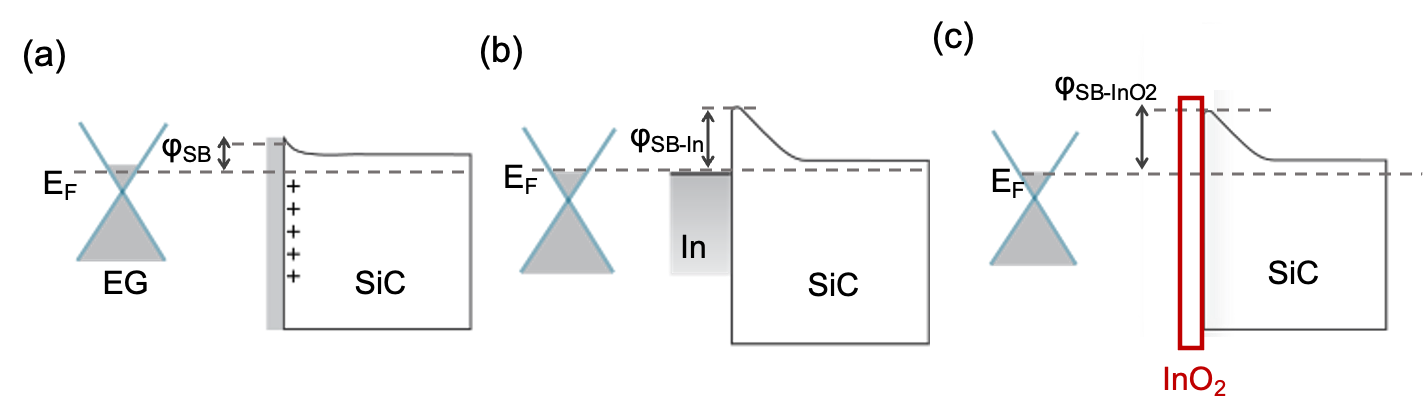


**Figure S21:** Simplified band diagrams of as-grown EG on SiC (a), In (b) and InO_2_ (c) intercalated EG/SiC, illustrating Schottky barrier formation via In/InO_2_ intercalation.


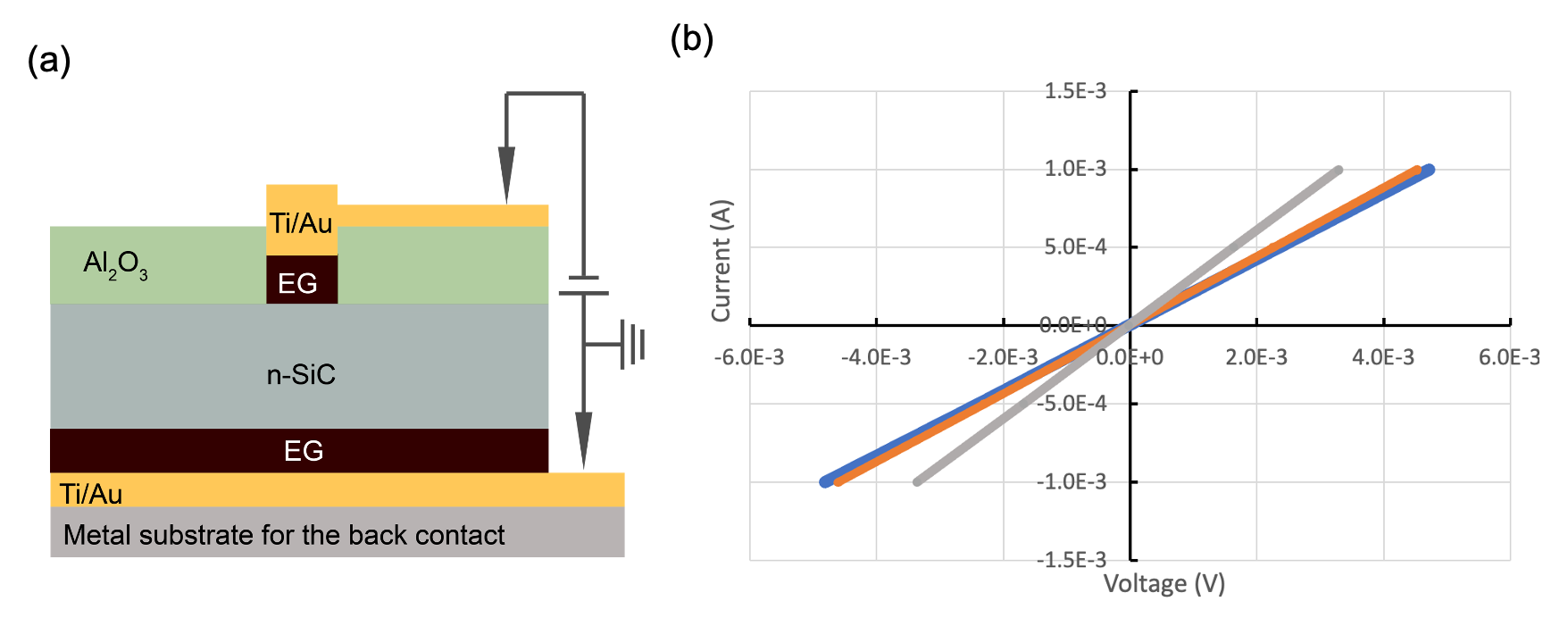


**Figure S22:** Two-terminal vertical device measurements with as-grown graphene/n-SiC contacted with Ti/Au. (a) showing the schematic for the measurement system. (b) I-V curves taken from Ti/Au contacted as-grown EG/n-SiC two terminal devices showing ohmic conduction.

The high leakage current observed under reverse bias (on the order of nA at –2 V) with EG/InO_2_/n-SiC diode occurs sporadically due to device-to-device variability. This variability arises mainly from spatial discontinuities of InO₂ on the nanometer scale. First, the continuity of the monolayer InO₂ is assessed using Auger electron spectroscopy (AES), which has a spatial resolution of ~100 nm under our data collection conditions using the Physical Electronics Versa Probe III. While AES confirms large-area coverage, nanoscale discontinuities in the intercalated InO₂ layer may exist and remain undetected unless examined with cross-sectional STEM (Figure S23). These discontinuities expose regions of the underlying EG/n-SiC interface, which is inherently ohmic due to the presence of the buffer layer. Such exposed regions act as low-resistance conduction paths, contributing to elevated leakage under reverse bias. Additionally, the step edges of the SiC substrate present a challenge for uniform intercalation due to their distinct crystallographic orientations. Although we intentionally fabricated devices on SiC terraces to minimize this effect, complete exclusion of step edges is difficult. These regions, like the buffer-layer EG/SiC interface, can also support ohmic conduction and serve as leakage pathways. These structural non-uniformities create localized discontinuities in the insulating barrier, explaining the observed leakage behavior beyond that expected from Fowler–Nordheim tunneling.


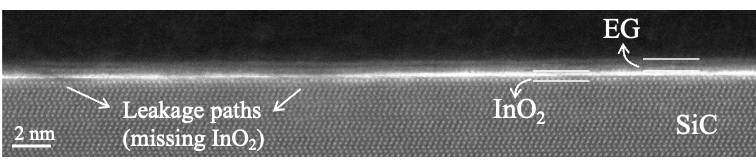


**Figure S23:** HAADF-STEM image of monolayer InO₂-intercalated EG, showing possible leakage paths for electrical transport where the InO₂ is missing at the interface.

Temperature dependent I-V (10 K – 400 K) was conducted on EG/InO_2_/n-SiC MOS-based Schottky diode to understand the vertical conduction mechanism and extract the barrier height, $\varphi_{B}$. The fit of the reverse bias current at 10 K with Fowler Nordheim Tunnelling (FNT) demonstrates a linear relationship (ln (I/V^2^) vs 1/V), verifying tunneling as dominant conduction mechanism for <150 K (Figure S21a). In the forward bias, the I-V curve is examined with the thermionic emission (TE) model, based on the Equations 1-4 in the main text (see methods). After extracting the reverse saturation current *I_o_* from *ln (I)* vs *V* plot (inset in Figure 4b), Richardson plot has been drawn for 320 K – 400 K (Figure S21b), where Richardson Constant (*A^*^*) and barrier height ($\varphi_{B}$) is extracted as 139 Acm^−2^K^−2^ and 0.19 eV, respectively. Although extracted $A^{*}$ is close to the theoretical Richardson constant of 4H-SiC (146 Acm^−2^K^−2^), the barrier height is lower than expected.


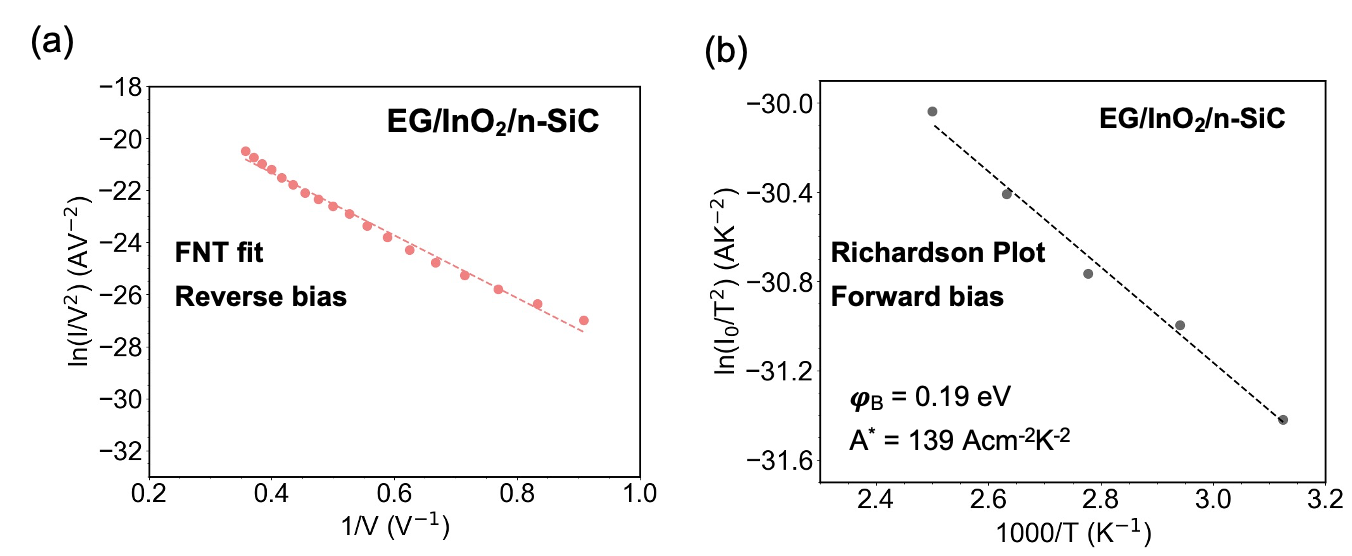


**Figure S24:** Fowler-Nordheim Tunneling (FNT) fit of the reverse bias current for EG/InO_2_/n-SiC vertical diode at 10 K, demonstrating linear relationship. Richardson plot for the forward bias current at 320 K – 400 K, where A^*^ and $\varphi_{B}$ are extracted as 139 Acm^−2^K^−2^ and 0.19 eV, respectively.

Alternatively, the barrier height at each temperature can be extracted from Equation 2 (main text) by using theoretical $A^{*}$ for 4H-SiC and device area (0.28 μm^2^) for 320 K – 400 K. As shown in Table S4, with higher temperatures, the barrier height increases from 0.39 eV to 0.49 eV and ideality factor reduces from 3.87 to 1.76. Such a temperature dependent behavior of $\varphi_{B}$ and $n$ is attributed to the inhomogeneity of the barrier height. At low temperature, electrons without sufficient energy can only surmount patches with lower Schottky barrier. As the temperature increases, more and more electrons gain sufficient energy to overcome higher barrier, leading to the measurement of higher apparent Schottky barrier height.^[11]^

**Table S4.** Reverse saturation current density, J_0_, barrier height, $\varphi_{B}$, and ideality factor, $n$, extracted from Equation 4 in the main text for the forward bias current for EG/InO_2_/n-SiC device. Increase in $\varphi_{B}$ and reduction in $n$ with temperature indicate barrier inhomogeneities at the EG/InO_2_/n-SiC interface.

| **Temperature**  **(K)** | ***J_0_*** | **Barrier height**  **(eV)** | **Ideality factor**  **(n)** |
| --- | --- | --- | --- |
| 320 | 10.49 | 0.39 | 3.87 |
| 340 | 5.89 | 0.44 | 2.82 |
| 360 | 8.94 | 0.45 | 2.18 |
| 380 | 20.29 | 0.45 | 2.16 |
| 400 | 14.88 | 0.49 | 1.76 |

The presence of barrier inhomogeneities is directly observed by conductive-AFM (C-AFM) measurements in both current mapping and I-V curves (Figure S22). Figure S22b presents several I-V curves taken from different regions on the EG/InO_2_/n-SiC sample, where reverse V_th_ changes between -4 V and -6 V. Additionally, in the C-AFM current mapping (Figure S22a), high conductivity regions are present, indicating barrier height inhomogeneities.


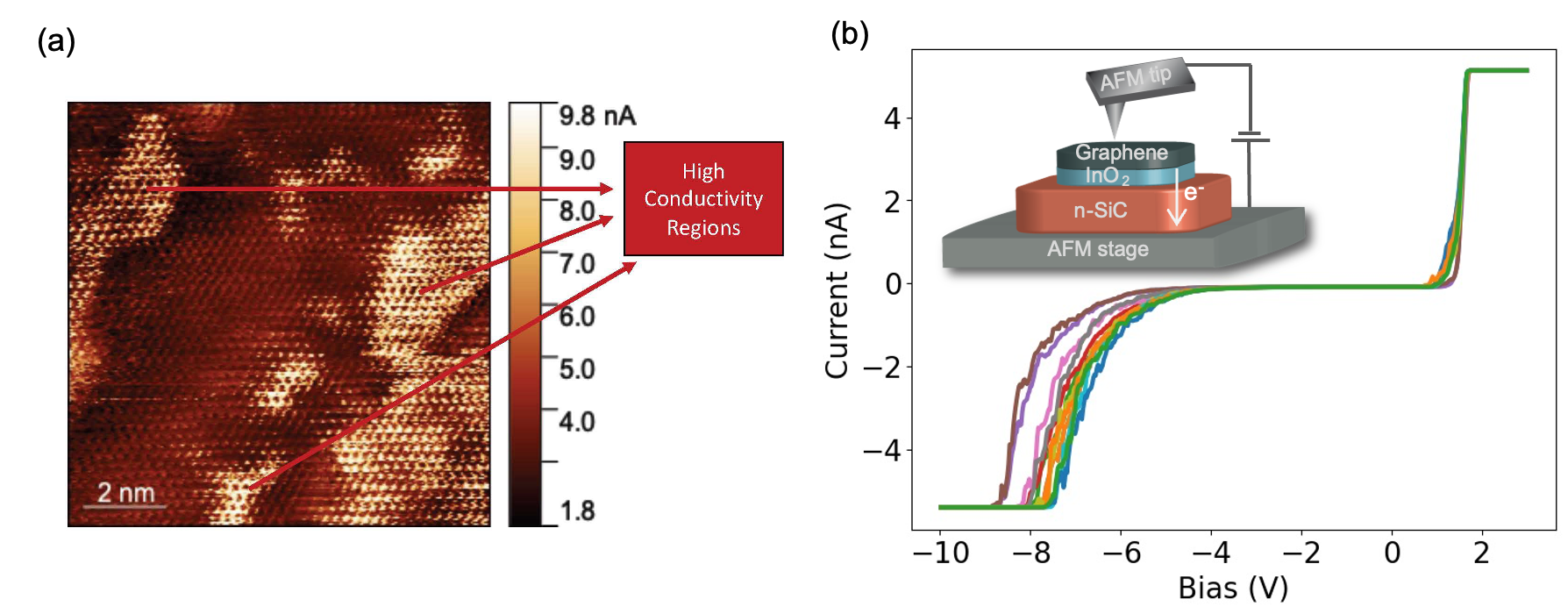


**Figure S25.** Nanoscale electrical characterization of the EG/InO_2_/n-SiC vertical diode via C-AFM. High conductivity regions are observed in the atomic-resolution current map in (a), indicating inhomogeneities in the barrier height. (b) Several I-V curves acquired from different regions on the sample, verifying variation in reverse leakage current with V_th_ changing between -4 V and -6 V.

# References

[1] C. Riedl, C. Coletti, T. Iwasaki, A. A. Zakharov, U. Starke, Structural and electronic properties of epitaxial graphene on SiC(0001): a review of growth, characterization, transfer doping and hydrogen intercalation, *Phys Rev Lett* **2009**, *103*, 1.

[2] N. Briggs, B. Bersch, Y. Wang, J. Jiang, R. J. Koch, N. Nayir, K. Wang, M. Kolmer, W. Ko, A. De La, F. Duran, S. Subramanian, C. Dong, J. Shallenberger, M. Fu, Q. Zou, Y. Chuang, Z. Gai, A. Li, A. Bostwick, C. Jozwiak, C. Chang, E. Rotenberg, J. Zhu, Atomically Thin Half-van der Waals Metals Enabled by Confinement Heteroepitaxy, *Nat. Mater.* **2020**, 19, 637.

[3] M. T. Wetherington, F. Turker, T. Bowen, A. Vera, S. Rajabpour, N. Briggs, S. Subramanian, A. Maloney, J. A. Robinson, 2-Dimensional Polar Metals: A Low-Frequency Raman Scattering Study, *2d Mater.* **2021**, 8, 041003.

[4] N. Sheremetyeva, D. Tristant, A. Yoshimura, J. Gray, L. Liang, V. Meunier, First-principles study of the thermodynamic and vibrational properties of ReS2 under pressure, *Phys. Rev. B* **2019**, *100*, 214101.

[5] N. Sheremetyeva, M. Lamparski, L. Liang, G. Borin Barin, V. Meunier, Resonant Raman in Armchair Graphene Nanoribbons from First-Principles, Carbon NY **2024**, 227, 119164.

[6] S. Sänze, C. Hess, Ethanol Gas Sensing by Indium Oxide: An Operando Spectroscopic Raman-FTIR Study, *Journal of Physical Chemistry C* **2014**, 118, 25603.

[7] D. B. Buchholz, Q. Ma, D. Alducin, A. Ponce, M. Jose-Yacaman, R. Khanal, J. E. Medvedeva, R. P. H. Chang, The Structure and Properties of Amorphous Indium Oxide, *Chemistry of Materials* **2014**, *26*, 5401.

[8] J. H. Klootwijk, C. E. Timmering, Merits and limitations of circular TLM structures for contact resistance determination for novel III-V HBTs, *IEEE International Conference on Microelectronic Test Structures*, **2004**, pp. 247–252.

[9] A. V. Kuchuk, P. Borowicz, M. Wzorek, M. Borysiewicz, R. Ratajczak, K. Golaszewska, E. Kaminska, V. Kladko, A. Piotrowska, Ni-Based Ohmic Contacts to n-Type 4H-SiC: The Formation Mechanism and Thermal Stability, *Advances in Condensed Matter Physics* **2016**, 9273702.

[10] S. Hertel, D. Waldmann, J. Jobst, A. Albert, M. Albrecht, S. Reshanov, A. Schöner, M. Krieger, H. B. Weber, Tailoring the graphene/silicon carbide interface for monolithic wafer-scale electronics, Nat Commun. **2012**, 3, 957.

[11] R. T. Tung, Electron Transport at Metal-Semiconductor Interfaces: General theory, Phys. Rev. B 1992, 45, 23.
